# Promotion‐ or Prevention‐Focused Self‐Evaluation and the Preferential Pursuit of More Desirable Romantic Partners

**DOI:** 10.1111/jopy.70043

**Published:** 2025-12-16

**Authors:** Eileen Z. Wu, Daniel C. Molden, Paul W. Eastwick

**Affiliations:** ^1^ Department of Psychology Northwestern University Evanston Illinois USA; ^2^ Department of Psychology University of California, Davis Davis California USA

**Keywords:** assortative mating, regulatory focus, relationship initiation, romantic desire

## Abstract

**Objective:**

This research examined how self‐regulatory orientations—promotion focus (growth) and prevention focus (security)—influence individuals' evaluation of their own desirable traits and their pursuit of highly desirable romantic partners.

**Method:**

Two studies analyzed participants' evaluations of themselves and romantic partners: Study 1 followed 208 college students longitudinally across 7 months of early romantic interest development; Study 2 observed 187 participants in live speed‐dating events. Participants' regulatory focus, self‐perceptions, partner ideals, and the desirability of pursued partners were assessed, controlling for self‐esteem and consensus ratings of participants' own desirability.

**Results:**

Stronger promotion concerns predicted overly positive self‐evaluations and pursuit of more desirable partners, whereas stronger prevention concerns predicted overly negative self‐evaluations and pursuit of less desirable partners. These effects persisted after accounting for self‐esteem and consensus ratings of participants' desirability and were partially mediated by participants' own self‐perceptions and partner ideals.

**Conclusions:**

Findings suggest that regulatory focus influences aspirations for highly desirable partners through exaggerations of positive or negative self‐perceptions that then influence aspirations for and willingness to pursue more or less subjectively and objectively desirable partners. Promotion and prevention mindsets thus appear to play a unique self‐regulatory role in romantic partner selection beyond general self‐esteem or objective desirability.

## 1 Introduction

When seeking a romantic partner, people encounter potential candidates with varying levels of overall desirability across a host of normatively desirable traits. Some individuals might have a more conservative estimate of who they can realistically attract and thus shift attention away from the more desirable partners, having decided that pursuing them is likely to bring only embarrassment and rejection. In contrast, other individuals might decide they possess enough romantic appeal to pursue partners who are more desirable. These reactions clearly reflect different levels of overall self‐confidence, but they also may reflect differences in fundamental motivations that influence people's evaluation and pursuit of possible romantic partners.

Although people tend to have a somewhat realistic sense of their desirability as a romantic partner and pursue relationships with others similar to themselves in desirability (e.g., Watson et al. 2004), such similarity effects are often modest (e.g., Shaw Taylor et al. 2011) or near zero (Kurzban and Weeden 2005; Luo and Zhang 2009; Tidwell et al. 2013; Wurst et al. 2018). In addition, many individuals do pursue more normatively desirable partners, even if not highly desirable themselves. The present research concerns how different motivational orientations might influence how people evaluate their own and others' romantic desirability, which could help explain when and why people pursue highly desirable partners. More specifically, we investigate how broad motivations for growth and advancement (i.e., *promotion*) versus for safety and security (i.e., *prevention*) predict people's interest in more desirable partners independent of their own level of desirability.

### 1.1 Valuing Desirable Traits in Romantic Partners

Research on romantic relationships has generally provided evidence for *assortative mating*, that is, that romantic couples systematically match on various characteristics, including demographics, physical traits, values, and personality (see Luo 2017 for a review). For example, studies of assortative mating in both dating and marital relationships have found consistent positive correlations between such characteristics (e.g., physical attractiveness; average *r* = 0.39 in the Feingold 1988 meta‐analysis). Various motivational (Buss and Shackelford 2008; Walster et al. 1978) and structural (Kalick and Hamilton 1986) explanations for matching effects have arisen, but, in addition, people also appear to prefer and pursue partners with more desirable traits independent of their own general level of desirability (e.g., Walster et al. 1966; Eastwick et al. 2014). Moreover, whatever realistic sense people have of their own desirability to potential partners, some may still place a greater or lesser priority on seeking highly desirable partners. That is, although everyone would like a highly desirable romantic partner, several factors influence how strongly people value overall desirability when pursuing such partners.

Some of these factors involved in pursuing highly desirable partners include general preferences and beliefs concerning romantic relationships. One example is how strongly people want to be in a romantic relationship (Spielmann et al. 2013); the fear of being single can drive people to settle for less desirable potential partners. Another example is the implicit theories people have about how relationships develop (Knee et al. 2001); believing that a relationship can grow and change could lead people to initially accept partners who are below their ideal desires in the hope things will improve, whereas believing that successful partnerships are destined to be could lead to stricter application of one's ideals for potential partners. Other factors involve people's general self‐worth and evaluations of their own desirability. For example, although both people of higher and lower self‐worth prefer dating partners with higher social desirability, people of higher self‐worth are more likely to contact these high‐desirability partners, presumably due to higher confidence in successful outcomes (Penke et al. 2007; Shaw Taylor et al. 2011). In the present research, we examine another potential factor in pursuit of and confidence in attaining more desirable romantic partners that involves broad self‐regulatory orientations. We propose that motivational concerns arising from such orientations can influence people's evaluation of their romantic appeal above and beyond their general self‐worth, as well as their willingness to risk rejection by identifying and pursuing more desirable romantic partners.

### 1.2 Motivations for Promotion or Prevention

A critical distinction in people's fundamental motivations has often been made between concerns with growth, advancement, and development versus concerns with security, safety, and protection (e.g., Bowlby 1969). Higgins's (1997) regulatory focus theory expands upon this distinction and describes how these respective concerns with *promotion* versus *prevention* also create different modes of self‐regulation that alter how people represent and pursue their goals (see Scholer et al. 2019).

When concerned with promotion, people strive to attain advancement and focus more on exploiting opportunities that bring them closer to their ideals. They see themselves as approaching growth (i.e., gains and the presence of positive outcomes) while avoiding missed opportunities (i.e., non‐gains and the absence of positive outcomes). In contrast, when concerned with prevention, people strive to maintain a desirable status quo and focus more on protecting against obstacles that might keep them from upholding their important standards. They see themselves as approaching security (i.e., non‐loss and the absence of negative outcomes) while avoiding threat (i.e., loss and the presence of negative outcomes). Note that these concerns with promotion or prevention are not equivalent to a focus on approach versus avoidance, respectively, as both concerns involve approaching desired outcomes (i.e., growth or security) and avoiding undesired outcomes (i.e., missed opportunities or threats). Instead, regulatory focus describes the motivational implications of attempting to draw closer to different types of desired outcomes and distance oneself from different types of undesired outcomes (for reviews see Higgins 1997; Molden and Winterheld 2013; Scholer et al. 2019).

Because promotion concerns center on the desired outcome of advancement, they create a more *eager* focus on seeking possible gains, even at the risk of losses. When promotion‐focused, people prioritize the possible missed opportunities of failing to pursue gains more than any setbacks that could result (e.g., Crowe and Higgins 1997; Molden 2012). In contrast, because prevention concerns center on the desired outcome of security, they create a more *vigilant* focus on limiting the possibility of loss, even at the risk of forgoing gains. When prevention‐focused, people prioritize the setbacks that could result from pursuing potential gains more than the missed opportunities caused by not pursuing such gains.^1^

An additional feature of the eager mindsets associated with promotion concerns involves reporting greater confidence during goal pursuit, which can help sustain eagerness. When promotion‐focused, people are (a) more strongly motivated by positive feedback and perceived progress (Förster et al. 2001; Idson and Higgins 2000), and (b) more likely to generate their own positive feedback, which includes holding more favorable self‐evaluations (Scholer et al. 2014) and preferring optimistic forecasts for future outcomes (Hazlett et al. 2011). In contrast, an additional feature of the vigilant mindsets associated with prevention concerns involves reporting less confidence during goal pursuit, which can help sustain vigilance. When prevention‐focused, people are (a) more strongly motivated by negative feedback and perceived lack of progress, and (b) more likely to generate their own negative feedback, which includes holding less favorable self‐evaluations and preferring more defensively pessimistic forecasts for future outcomes.

### 1.3 Motivations for Promotion or Prevention and the Pursuit of Desirable Romantic Partners

When focused on identifying and pursuing potential romantic partners, targeting a higher level of desirability could potentially be seen either as an ideal to strive toward or an important standard that must be maintained. That is, pursuing a romantic partner with more desirable qualities overall could help maximize feelings of either growth or security. However, at this initial stage of selecting among romantic options, although pursuing highly desirable individuals could create greater opportunities for these valued outcomes, it also entails greater risks of setbacks and loss (i.e., a higher likelihood of an unpleasant rejection by such partners, e.g., Montoya 2008). Therefore, although partners with higher overall levels of desirability do not inherently better serve to fulfill promotion or prevention concerns, when focused on promotion, people might more eagerly consider the romantic opportunities pursuing highly desirable partners could provide despite the risks and be more likely to seek them out, whereas when focused on prevention, people might more vigilantly consider the interpersonal risks of pursuing highly desirable partners despite the potential rewards and be less likely to seek them out.

Previous research by Hui et al. (2020) has provided initial evidence for these types of influences of promotion and prevention concerns on pursuit of highly desirable partners. These studies found that stronger chronic or experimentally‐induced promotion concerns predicted stronger stated desires to seek a romantic partner more physically attractive than oneself. In addition, among individuals who had successfully formed a romantic relationship, stronger promotion concerns predicted having an existing partner high in physical attractiveness, even though these motivations were not associated with being more physically attractive oneself. These results are thus consistent with the hypothesis that promotion concerns motivate strategies of eagerly seeking the perceived opportunities of pursuing a more desirable partner even if this ultimately might lead to higher chances of rejection.

Beyond these types of effects, as outlined above, there is an additional, complementary route through which regulatory focus might influence the pursuit of highly desirable romantic partners. When evaluating romantic partners, the more attractive and desirable people consider themselves, and the greater positive expectations they have about successfully pursuing desirable partners, the higher they might set their aspirations (Penke et al. 2007; Shaw Taylor et al. 2011). Thus, the more favorable self‐perceptions associated with promotion concerns and eager mindsets could reduce perceived discrepancies between oneself and highly desirable partners, leading to stronger preferences for such partners independent of one's own actual desirability. However, the less favorable self‐perceptions associated with prevention concerns and vigilant mindsets could highlight perceived discrepancies between oneself and highly desirable partners, leading to weaker preferences for such partners independent of one's own actual desirability. That is, beyond any cognitive mechanisms of self‐perception or the adjustment of self‐evaluations based on individual histories of romantic success (e.g., Beckage et al. 2009), there could be additional self‐regulatory mechanisms that influence how high people set their aspirations when pursuing romantic partners.

Therefore, extending the previous studies by Hui et al. (2020), the current research seeks to develop a fuller picture of the mechanisms by which regulatory focus might predict selection and pursuit of romantic partners through the influence of people's more positive or negative perceptions of their own desirability on their pursuit of desirable partners. In addition, this research also expands Hui et al.'s analysis beyond desires for physically attractive partners and includes other desirable traits a romantic partner might more generally possess.

### 1.4 The Present Studies

Study 1 first examined how regulatory focus predicted participants' perceptions of both their own positive traits and the traits they would want in an ideal partner. This study further assessed how such perceptions predicted the reported desirability of the specific individuals toward whom participants expressed romantic interest over the following 7 months. Study 2 next evaluated how regulatory focus predicted participants' assessment of their own positive traits and ideal‐partner traits and, in turn, how these assessments predicted the desirability of the traits possessed by speed‐dating partners participants expressed interest in seeing again. In both studies, to further examine how associations of regulatory focus with these outcomes were independent of other individual differences that could influence the value placed on desirability in romantic partners, additional measures of gender and self‐esteem were included across these studies. In addition, to evaluate whether associations of regulatory focus truly reflected eager or vigilant mindsets rather than accurate perceptions of how others value them, more objective measures of participants' own traits, as evaluated by the consensus of their speed‐dating partners, were also included in Study 2.

If promotion concerns are associated with eager, more positive views of oneself and prevention concerns are associated with vigilant, less positive views of oneself, this leads to the following hypotheses: First, promotion concerns should predict higher, and prevention concerns should predict lower ratings of one's own positive traits (Scholer et al. 2014). As outlined earlier, higher or lower ratings of one's own qualities should then further predict overall levels of aspiration in the higher or lower ratings of desired traits in an ideal romantic partner, which then, in turn, should predict higher or lower ratings of the positive traits of specific individuals identified as potential partners and pursued for possible relationships (see Penke et al. 2007; Shaw Taylor et al. 2011). Thus, in addition to any direct effects of regulatory focus on aspiring to higher levels of desirability for hypothetical partners and on actively pursuing more desirable romantic prospects (see Hui et al. 2020), if the hypothesized eager or vigilant self‐perceptions associated with regulatory focus also play a role, there should also be indirect effects of regulatory focus on preferring more desirable partners through self‐perceptions and ideal standards.

Given these hypotheses, the primary focus of the present analyses is on overall levels of aspiration and the broad normative desirability of the romantic partners that people identify and pursue, rather than on matches between people's specific romantic ideals and attraction to particular partners (i.e., whether people who ideally desire a caring partner are uniquely attracted to and pursue individuals who are more caring, see Eastwick et al. 2019). One reason for this focus is that, although it is still a topic of debate, previous research has questioned whether people's own unique ideals robustly predict their romantic partner preferences above and beyond normative desirability (i.e., whether any correspondence between ideal desires for caring partners and pursuit of such partners is explained by anything other than the general positivity people assign to being caring, see da Silva Frost and Eastwick 2024; Sparks et al. 2020). Another more immediate reason is that, as detailed below, across the set of traits used in the present studies, participants' ratings, whether of themselves or others, on the set of positive traits provided were highly intercorrelated (*α*s = 0.66–0.88), suggesting a strong consensus in the desirability of these traits rather than distinct preferences for particular traits in these samples. For these reasons, although it is theoretically possible to posit hypotheses about specific desirable traits in a relationship partner that could be uniquely associated with concerns with promotion (i.e., qualities promising growth and enjoyment) or prevention (i.e., qualities promising stability and security; see Cortes et al. 2018; Hui et al. 2013; Molden and Finkel 2010), such unique preferences are not the focus of the present analysis of relationship initiation. However, for those interested in such hypotheses, in the Figures S6, S7, S11, and S12, we detail separate analyses of distinct vitality and passion‐oriented traits, potentially of greater appeal when concerned with growth and promotion, versus trustworthiness and loyalty‐oriented traits, potentially of greater appeal when concerned with security and prevention (see Fletcher et al. 1999).

## 2 Study 1

Study 1 examined how associations of regulatory focus with confidence in one's own desirable traits as a romantic partner might further predict (a) the stated importance assigned to a potential partner's positive traits in a decision to start a romantic relationship and (b) the reported positive traits of specific individuals people identified as their current romantic interests as part of a longitudinal study of early relationship development (see Eastwick et al. 2023).

### 2.1 Method

#### 2.1.1 Participants and Procedures

No observation was excluded, and all relevant measures were reported in these studies. Participants were 208 college students from a private university in the Midwestern United States, the maximum number of participants that could be recruited with the resources available. They all identified as heterosexual and single and were recruited within 3 weeks of the start of their first year of college to participate in a longitudinal study (91 men, 117 women; age: *M* = 18.11, SD = 0.31). The sample was 64% White, 21% Asian, 4% African American, and 3% multi‐racial, with 2% who identified their race as “other”; in addition, 10% of the sample reported Hispanic, Latino, or Spanish ethnicity. After completing an initial online questionnaire including personality, relationship history, and relationship preference measures, participants attended an in‐person lab session, where they completed additional relationship background measures. Then, participants completed 10 waves of an online questionnaire about their current romantic interests once every 3 weeks, for a total study length of approximately 7 months. At the end, participants were debriefed and paid up to $100 depending on how many waves they completed. On average, participants completed 9.3 (SD = 1.8) waves, with 171 (83%) completing all waves.

#### 2.1.2 Materials

##### 2.1.2.1 Measuring Regulatory Focus and Self‐Perceptions

The initial online questionnaire and the first lab session included a variety of individual difference measures and self‐ratings. Participants completed the Regulatory Focus Questionnaire (RFQ) to measure general concerns with promotion and prevention (Higgins et al. 2001). This questionnaire assesses people's subjective histories of success with eager and vigilant strategies of self‐regulation and thus serves as a proxy for their tendency to further employ each type of strategy. **S**ix items measure promotion histories (α = 0.71; e.g., “when it comes to achieving things that are important to me, I find that I don't perform as well as I ideally would like to do” [reverse scored]) and five items measure prevention histories (α = 0.79; e.g., “not being careful enough has gotten me into trouble at times [reverse scored]”). The scale is well‐validated and has strong predictive validity in a variety of domains, including romantic relationships (e.g., Bohns et al. 2013; Cortes et al. 2018; Hui et al. 2013; Molden and Finkel 2010; see Molden and Winterheld 2013). It also has good discriminative validity; correlations with big five personality traits are low to moderate (Gorman et al. 2012), and, as would be expected from measures of perceived success, both promotion and prevention histories have small positive associations with achievement motivation, planning, and active coping (Grant and Higgins 2003). Because of the focus in the present research on the downstream impact of positive self‐impressions, participants also completed Rosenberg's (1965) global self‐esteem scale (α = 0.92) to directly assess whether any observed associations of promotion and prevention concerns with the pursuit of desirable romantic partners could be attributed to the additional association of these concerns with overall self‐esteem.^2^

To measure perceptions of their own positive traits, participants rated their agreement that a series of positive, desirable traits described their “actual self”. The traits were “physically attractive”, “ambitious/driven”, “confident”, “exciting”, “spontaneous” “dependable”, “supportive”, “level‐headed”, “optimistic”, “realistic”, “creative”, and “patient”.^3^ Although not originally chosen with such criteria in mind, note that traits potentially related more to growth and promotion (e.g., exciting, spontaneous, confident, optimistic) and traits potentially related more to security and prevention (e.g., dependable, supportive, level‐headed, patient) were both represented. Ratings across all traits were moderately intercorrelated (α = 0.66). All these measures were answered on 1 (*Strongly disagree*) to 7 (*Strongly agree*) scales.

##### 2.1.2.2 Measuring Preferred and Reported Desirability of Romantic Interests

To assess ideas about participants' ideal romantic partner, at the first lab session, they rated whether the same traits they evaluated themselves on “matter[s] in your decision to start a romantic relationship with someone”. The importance ratings across these traits were again intercorrelated (α = 0.76). Then, at each online wave, participants listed the “… two people you've met since coming to [college] with whom you are most interested in forming a romantic relationship”; they could repeat interests across waves, and, on average, listed 5.13 (SD = 2.14) unique individuals over the course of the study. Whenever a new interest was listed, whether at the first or any subsequent wave, participants rated whether their romantic interests possessed the same set of traits on which they had rated themselves and their ideal partner at the outset. Ratings of romantic interests across these traits were once again consistently intercorrelated (α = 0.84) and were again made on 1 (*Strongly disagree*) to 7 (*Strongly agree*) scales. Also see Table S1 for evidence that participants were not normatively weighing a specific or smaller collection of traits more heavily than others, which supports aggregating trait ratings into a single index of desirability.

### 2.2 Results

**FIGURE 1:**
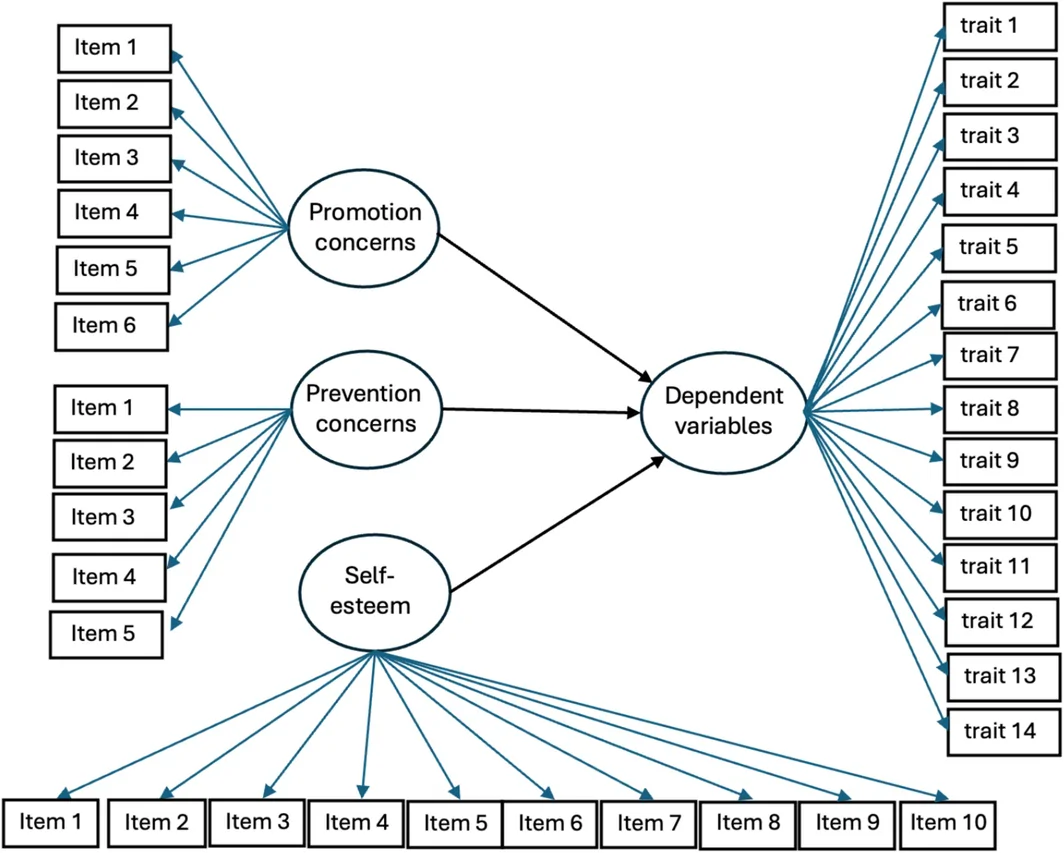
A schematic representation of the latent variable models used to test associations of promotion and prevention concerns with the outcomes of interest. All SEM analyses conducted included covariances between the latent variables for promotion concerns, prevention concerns, and self‐esteem. These pathways are left out of the schematic to improve readability.

Means, SDs, and zero‐order correlations between all person‐level measures in Study 1 are presented in Table 1. To ensure optimal tests of the association of regulatory focus with participants' perceptions of themselves, preferences for desirable partners, and reports of their identified partners above and beyond associations with overall self‐esteem, all analyses were performed using structural equation modeling (SEM) with latent variables. This approach directly models the measurement error in the independent and dependent variables and reduces the bias such error can create in traditional multiple regression approaches (see Culpepper and Aguinis 2011; Wang and Eastwick 2020; Westfall and Yarkoni 2016). Repeating the analyses with more traditional regression approaches leads to highly similar conclusions, and details of this alternative approach can be found in the supplement (Table S2). After initial confirmatory factor analyses (CFA) supporting that promotion and prevention concerns represented separate constructs distinct from self‐esteem (see Supporting Information for details, 4), latent indicators of these constructs were used to predict latent indicators of the dependent variables of interest (see the schematic in Figure 1). These models were estimated using the lavaan package in R (Rosseel 2012). All SEM models were fitted using unweighted least squares (ULS), which performs better than maximum likelihood estimation in smaller samples with ordinal data (Li 2016).

**TABLE 1:** Means, standard deviations, and zero‐order correlations of the measures (Study 1).

	Mean (SD)	1	2	3	4	5
1. Promotion	5.53 (0.80)	—				
2. Prevention	4.54 (1.25)	0.08	—			
3. Self‐esteem	5.35 (1.08)	0.66***	0.06	—		
4. Perceptions of own desirable traits	5.27 (0.59)	0.56***	0.02	0.60***	—	
5. Importance of desirable traits in an ideal partner	5.66 (0.53)	0.19**	−0.01	0.12^†^	0.40***	—
6. Perceived desirable traits of one's specific romantic interests	5.43 (0.48)	0.20**	−0.03	0.18**	0.37***	0.44***

*Note:* All variables were measured on 1 (strongly disagree)–7 (strongly agree) scales.

^†^ *p* < 0.10.

** *p* < 0.01.

*** *p* < 0.001.

**FIGURE 2:**
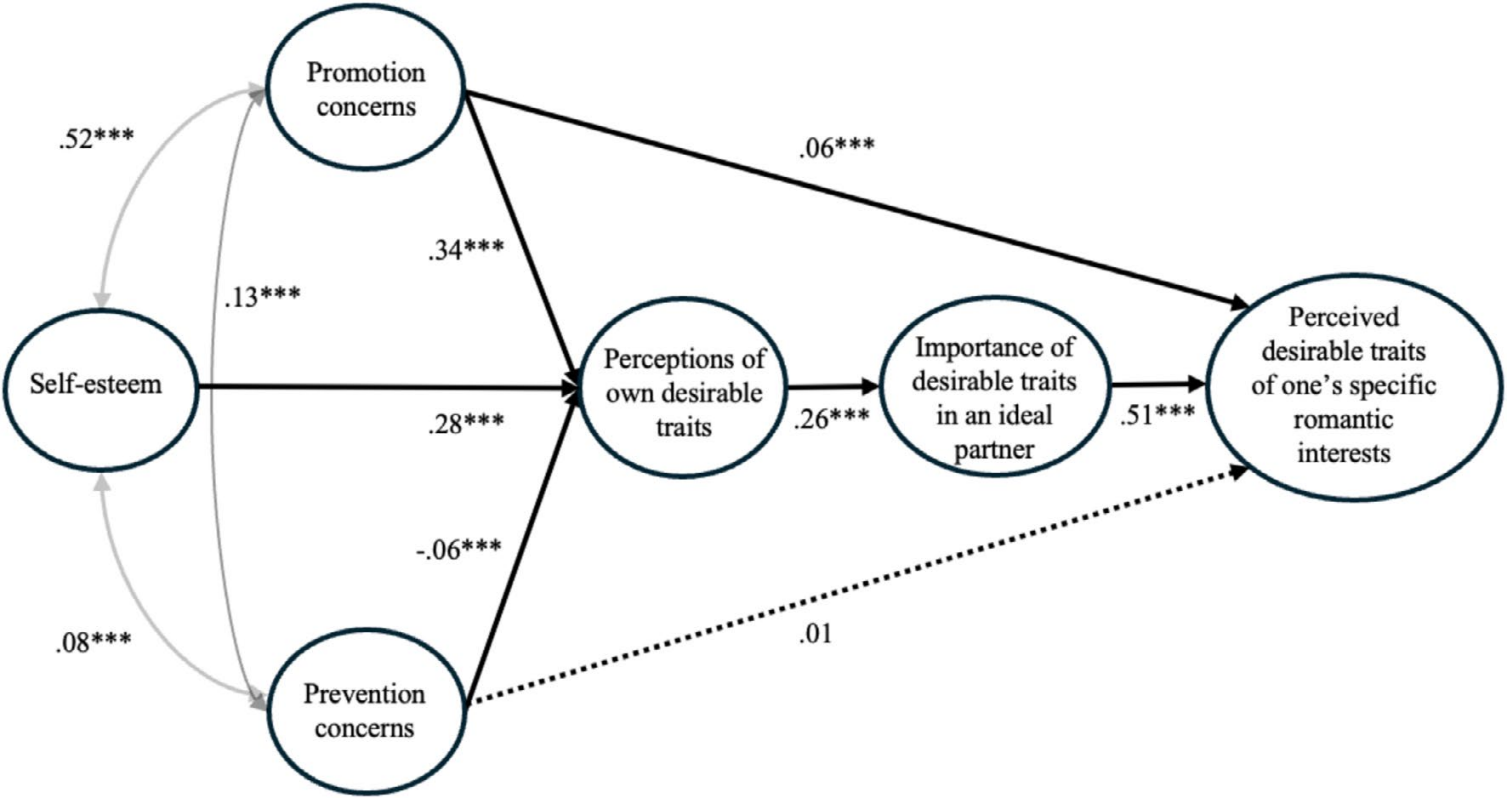
Perceptions of romantic interests' desirable traits as predicted by promotion and prevention concerns, perceptions of one's own desirable traits, and rated importance of desirable traits when seeking a romantic partner (Study 1). Coefficients represent unstandardized estimates from latent variable regressions. Ratings of each trait of participants' specific romantic interests were averaged across all the individuals they nominated over the course of the study. Overall, indirect effect of promotion: *z* = 5.17, *p* < 0.001, *b* = 0.049, 95% CI [0.030, 0.067] Overall, indirect effect of prevention: *z* = −7.02, *p* < 0.001, *b* = −0.010, 95% CI [−0.012, −0.007]. ***p < 0.001.

To examine primary hypotheses about the associations of participants' promotion and prevention concerns with their perceptions of their own desirable traits, and the subsequent associations of these perceptions with ratings of preferences for romantic partners and the ratings of their specific romantic interests, we assessed the SEM model depicted in Figure 2. In this model, perceptions of one's own desirable traits and importance ratings of desirable traits in an ideal partner were treated as serial mediators predicting reports of desirable traits in the specific romantic interests nominated over the 7 months of the study, averaged across all these individual interests. To assess additional associations of regulatory focus with preferences for romantic partners beyond self‐perception mechanisms, direct pathways between promotion and prevention concerns and ratings of specific romantic interests were also included in the model. Finally, participants' self‐esteem was included in the model as a control variable predicting perceptions of one's own desirable traits, as were covariances between esteem and regulatory focus.

The overall fit of the SEM model was slightly below typically acceptable standards, CFI = 0.87, RMSEA = 0.12. Exploratory analyses of the source of the poor fit indicated additional covariances between subsets of the specific traits used to construct the latent variables for self‐perceptions, romantic partner ideals, and ratings of romantic interests. For the main analyses, rather than risk capitalizing on chance inter‐trait associations within the different samples of Studies 1 and 2, we chose to retain the simplest structure for the latent estimates of all dependent variables (as depicted in Figure 1). However, to ensure our conclusions did not entirely rest on poor‐fitting models, we performed a post hoc examination of the modification indices for the model in Figure 2 and added additional covariances between specific questionnaire items that constituted the latent variables to achieve an acceptable fit. These analyses, reported in the supplement (Figure S1), did not alter the significance of any of the focal relationships between the latent variables.

Consistent with the hypotheses stated at the outset, the results shown in Figure 2 indicate that promotion concerns had a significant positive indirect effect on reports of the desirable traits possessed by one's specific romantic interests, *z* = 5.17, *p* < 0.001, *b* = 0.049, 95% CI [0.030, 0.067]. In contrast, prevention concerns had a significant negative indirect effect on these reports, *z* = −7.02, *p* < 0.001, *b* = −0.010, 95% CI [−0.012, −0.007].^4^ In addition, consistent with Hui et al. (2020), there was an additional positive direct effect of promotion concerns, but not of prevention concerns, on reports of desirable traits possessed by specific romantic interests. Comparisons with an alternative model in which the direct effects of regulatory focus were omitted and only the indirect effects remained had significantly poorer fit to the data, *χ* ^2^ = 36.8, df = 2, *p* < 0.001.

To evaluate moderation by gender, an alternate SEM model based on Figure 2 was estimated in which the regulatory focus associations were allowed to vary between men and women. This new model was then directly compared to a model in which these pathways were constrained to be the same across gender to test for moderation. Results showed that the new model had better overall fit, *χ* ^2^ = 10.2, df = 4, *p* = 0.04, suggesting the size of the regulatory focus associations was not the same for men and women. Follow‐up analyses revealed that the absolute magnitude of the regulatory focus associations with perceptions of own desirable traits and the direct associations of promotion concerns were smaller for men than women, but were still statistically significant for both groups, as were the overall indirect effects. Additional details of these results can be found in the supplement (Figures S2 and S3).

### 2.3 Discussion

Study 1 showed preliminary evidence that, as in past work (Scholer et al. 2014), promotion concerns were associated with higher perception of one's own desirable traits, whereas prevention concerns were associated with lower perception of one's own desirable traits. These perceptions then further predicted how high a level of aspiration they set for the desirability of an ideal romantic partner and the specific reported desirability of one's current romantic interests, even when controlling for self‐esteem.^5^ In addition to these indirect effects of regulatory focus through self‐perceptions, there was also evidence of additional direct effects of promotion concerns on higher reports of desirable traits for current romantic interests. Thus, Study 1 provided some initial support for both the complementary indirect and direct influences of regulatory focus on seeking highly desirable partners discussed at the outset.

However, one limitation of this first study is that it is not possible to determine whether our findings were due to individuals' promotion and prevention concerns respectively predicting overly positive or negative outlooks of their own traits, consistent with eager or vigilant mindsets, or whether individuals who report these stronger concerns would actually be rated by others as being higher or lower in desirable traits overall. Study 2 included consensus ratings of participants' traits by potential romantic partners to address this limitation. In addition, participants' reports of the traits of specific romantic interests might be biased to conform to their own ideals (Eastwick et al. 2019). Therefore, Study 2 also included consensus ratings of desired romantic partners' actual traits to provide a clearer measure of how higher standards for ideal partners might predict pursuit of objectively more desirable individuals.

## 3 Study 2

Study 2 conceptually replicated Study 1 by investigating how regulatory focus predicted single individuals' ideals for and perceptions of desirable traits in potential romantic partners they met during a live interaction. As part of a larger study on speed‐dating, participants had a series of four‐minute interactions with approximately 12 potential romantic partners and, after each one, rated their perceptions of these individuals' traits and how much they liked them. At the end of the study, participants then further decided who among these individuals they would be interested in seeing again.

### 3.1 Method

#### 3.1.1 Participants and Procedures

No observation was excluded, and all relevant measures were reported in these studies. Participants were 187 college students from a private Midwestern university. They all identified as heterosexual and single and were recruited to take part in one of 12 speed‐dating sessions with equal numbers of men and women (93 women, 94 men; age: *M* = 19.6, SD = 1 year). Overall, the sample was 69% White/Caucasian, 15% Asian/Asian American, 3% Hispanic, 2% African American, 3% Middle Eastern, and 8% other races or mixed races.

After completing a 30‐min online intake questionnaire that included personality, relationship history, and relationship preference measures, participants reported to a speed‐dating session approximately 11 days later. During the session, they had 4‐min speed‐dates with 11 or 12 opposite‐sex participants (depending on event attendance) in a round‐robin format and, immediately after each one, rated their speed‐dating partner on a two‐minute interaction‐record questionnaire. After the session, in an online “matching” survey, participants rated who among all the people they met they would like to see again, with the understanding that if a particular partner also wanted to see them again, they would receive that person's contact information. Participants also answered follow‐up questions about this decision. Everyone attended the speed‐dating events without cost and was paid $5 at the end of the event for agreeing to complete the related questionnaires.^6^

#### 3.1.2 Materials

##### 3.1.2.1 Measuring Regulatory Focus and Self‐Perceptions

As in Study 1, in the initial online questionnaire before the speed‐dating event, participants completed a variety of individual difference measures and self‐ratings. Among these were the same measures of promotion (α = 0.74) and prevention (α = 0.80) concerns, and a three‐item measure of global self‐esteem (“I have high self‐esteem”, “at times I think I am no good at all”, “on the whole, I am satisfied with myself.”; α = 0.83), all measured on the 1–7 scales used previously. Participants also completed ratings of their own desirable traits by reporting their agreement that a series of traits described their “actual self”. The traits were “physically attractive”, “sexy/hot”, “ambitious/driven”, “fun/exciting”, “funny”, “responsive”, “confident”, “assertive”, “good career prospects”, “charismatic”, “smart”, “intellectually sharp”, “dependable/trustworthy”, and “friendly/nice”. Again, although not originally chosen with such criteria in mind, traits potentially related more to growth and promotion (e.g., fun/exciting, confident) and traits potentially related more to security and prevention (e.g., responsive, dependable/trustworthy) were both represented. Ratings across these specific traits were highly intercorrelated (α = 0.86), and all these measures were answered on 1 (*Not at all*) to *9* (*Extremely*) scales.

##### 3.1.2.2 Measuring Preferred, Reported and Actual Desirability of Potential Romantic Partners

In the initial online questionnaire, participants rated the extent to which the same set of traits described their “ideal romantic partner” on a 1 (*Not at all*) to 9 (*Extremely*) scale. Ratings across these ideal traits were also highly intercorrelated (α = 0.88). After each speed‐date, all participants rated on the interaction‐record questionnaire the extent to which they thought their partner possessed these traits on a 1 (*Not at all*) to 9 (*Extremely*) scale. In addition, because everyone in each speed‐dating session ended up rating all other members of that session, it was possible to create consensus measures of each participant's traits by averaging the responses on these items across the 11 or 12 other participants with whom they interacted. Across all participants in the sample, this consensus represented generally reliable measures of “objective” ratings for each of the separate traits (αs > 0.68, with the exception of the trait dependable/trustworthy, which was α = 0.55). The consensus ratings across all of the different traits were also highly intercorrelated (α = 0.89). Also see Table S4 for evidence that participants were not normatively weighing a specific or smaller collection of traits more heavily than others, which supports aggregating trait ratings into a single index of desirability.

### 3.2 Results

Means, SDs, and zero‐order correlations between measures in Study 2 are presented in Table 2. After initial CFA again indicated that promotion and prevention concerns represented separate constructs distinct from self‐esteem (see Supporting Information for details, 19), SEM models with latent variables were used to estimate the associations of promotion and prevention concerns with the dependent variables of interest (see Figure 1). As before, these models were estimated with the R package lavaan and utilizing ULS.

**TABLE 2:** Means, standard deviations, and zero‐order correlation matrix (Study 2).

	Mean (SD)	1	2	3	4	5	6	7
1. Promotion	5.27 (0.91)	—						
2. Prevention	4.76 (1.24)	0.27***	—					
3. Self‐esteem	5.01 (1.26)	0.54***	0.08	—				
4. Perception of own desirable traits	5.98 (0.66)	0.68***	0.07	0.62***	—			
5. Own desirable traits by consensus	6.90 (0.83)	0.29***	0.13^†^	0.19**	0.23**	—		
6. Preferred trait desirability of ideal romantic partner	7.79 (0.73)	0.32***	0.04	0.24***	0.45***	0.02	—	
7. Consensus trait desirability of pursued speed‐dates	6.30 (0.38)	0.22**	0.01	0.17*	0.13^†^	−0.04	0.26***	—
8. Consensus trait desirability of not pursued speed‐dates	5.71 (0.27)	0.13^†^	0.00	0.04	0.12	−0.03	0.00	0.27***

*Note:* Promotion concerns, prevention concerns, and self‐esteem were measured on 1–7 scales. Perceptions of own desirable traits, desirable traits in ideal partner, and consensus trait desirability of pursued and not pursued speed‐dates were measured on a 1–9 scale.

^†^ *p* < 0.10.

* *p* < 0.05.

** *p* < 0.01.

*** *p* < 0.001.

**FIGURE 3:**
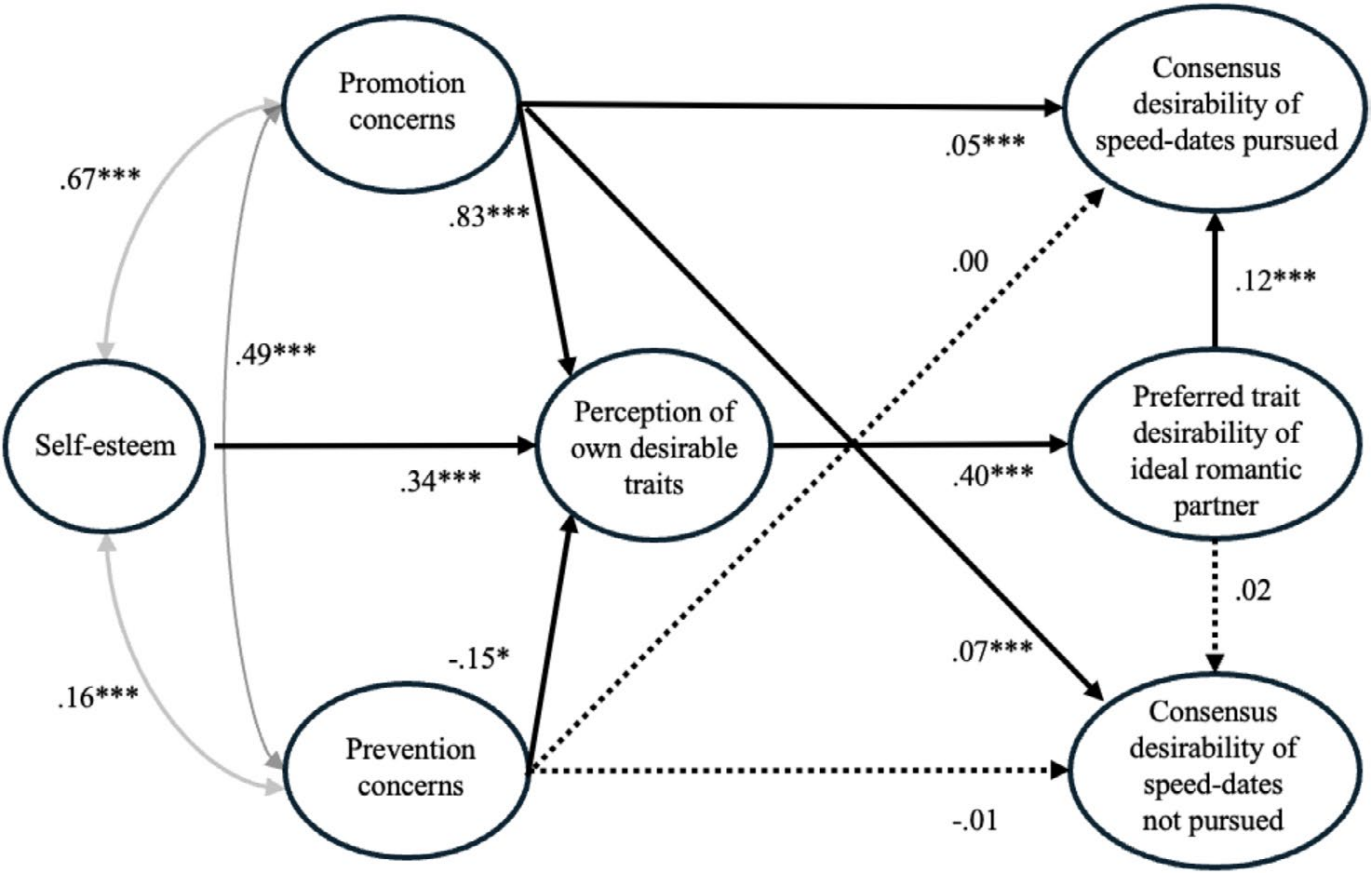
Consensus desirability ratings of partners participants did or did not pursue as predicted by promotion and prevention concerns, perceptions of one's own desirable traits and the desirable traits of an ideal romantic partner (Study 2). Coefficients represent unstandardized estimates from latent variable regressions. A latent variable of the consensus ratings of participants' own desirable traits by all speed‐dating partners was also included as a covariate predicting participants' perceptions of these traits (not depicted). Consensus ratings of speed‐dating partners participants did or did not pursue romantic were averaged separately across all of the individuals in these categories. Overall, indirect effect of promotion: *z* = 5.77, *p* < 0.001, *b* = 0.039, 95% CI [0.026, 0.052]. Overall, indirect effect of prevention: *z* = −5.27, *p* < 0.001, *b* = −0.010, 95% CI [−0.010, −0.004]. *p < 0.05, ***p < 0.001.

To examine primary hypotheses about the association of participants' promotion and prevention concerns with their overly positive or negative perceptions of their own desirable traits, and the subsequent associations of these perceptions with ideals for romantic partners and pursuit of partners with desirable traits, we assessed the SEM model depicted in Figure 3. In this model, perceptions of one's own desirable traits and ratings of desirable traits in ideal partner were treated as serial mediators predicting the consensus ratings of the desirable traits of the speed dates participants chose to pursue, as well as the consensus ratings of the desirable traits of the speed dates participants chose not to pursue. Repeating the analyses with more traditional regression approaches leads to highly similar conclusions, and details of this alternative approach can be found in the supplement (Table S5).

The consensus ratings of the individual traits for each speed‐dating partner participants met were separately averaged across the partners participants chose to pursue (i.e., indicated they wanted to see again) and the partners participants chose not to pursue (i.e., did not indicate they wanted to see again). As in Study 1, to assess additional associations of regulatory focus with preferences for romantic partners beyond self‐perception mechanisms, direct pathways between promotion and prevention concerns and consensus ratings of both pursued and not pursued partners were also included in the model. Finally, participants' self‐esteem was also included in this model as a control variable predicting perceptions of one's own desirable traits along with covariances between esteem and regulatory focus, along with consensus ratings of participants' own desirable traits as a control variable predicting perceptions of one's own desirable traits, along with its covariances between self‐esteem and regulatory focus. Thus, the regulatory focus coefficients in Figure 3 represent the increased positivity or negativity of participants' perceptions of their own traits beyond how others perceived them and their own general self‐worth.

The overall fit of the SEM model was acceptable, CFI = 0.92, RMSEA = 0.075. Again, consistent with the hypotheses outlined at the outset, results indicated that promotion concerns had a significant positive indirect effect on the consensus desirability of the traits possessed by specific speed‐dating partners participants chose to pursue, *z* = 5.77, *p* < 0.001, *b* = 0.039, 95% CI [0.026, 0.052]. In contrast, prevention concerns had a significant negative indirect effect on the desirability of these partners, *z* = −5.27, *p* < 0.001, *b* = −0.010, 95% CI [−0.010, −0.004].^7^ The indirect effects on the consensus desirability of the traits possessed by specific speed‐dating partners participants chose not to pursue were not significant, *z*s < 1.20, *p*s > 0.26, |*b*|s < 0.01. Comparisons with an alternative SEM model that constrained the paths between ideal preferences and the consensus traits of both pursued and not pursued partners to be the same showed significantly poorer fit, *χ* ^2^ = 12.46, df = 1, *p* < 0.001, which provides evidence that participants' ratings of the overall desirability of their ideal partner were more strongly associated with the normatively desirability of the partners that they did versus did not pursue.

As in Study 1, there was an additional positive direct effect of promotion concerns, but not of prevention concerns, on the consensus desirability of partners participants chose to pursue. An additional, unexpected finding illustrated in Figure 3 is that there was also a positive direct effect of promotion concerns on the consensus desirability of partners participants chose not to pursue. This similar effect for all speed‐dating partners, whether pursued or not, suggests that, in this sample, participants with stronger promotion concerns were more likely to be part of speed‐dating sessions where individuals were, on average, rated as more desirable overall. Because a comparison with an alternative SEM model in which the direct effect of promotion concerns on the desirability of chosen and non‐chosen partners was constrained to be equivalent did not reveal any worse fit, *χ* ^2^ = 0.43, df = 1, *p* = 0.51, the observed direct effects cannot be unambiguously interpreted as further support for extending the findings of Hui et al. (2020) to broader ratings of partner desirability.

As in Study 1, to evaluate any influence of gender on these results, an alternate SEM model was also estimated in which the regulatory focus regression pathways were allowed to vary between men and women. This new model was then directly compared to a model in which these regression pathways were constrained to be the same across gender to test for moderation. Results showed that the new model had better overall fit, *χ* ^2^ = 56.10, df = 6, *p* < 0.001, suggesting the size of the regulatory focus associations was not the same for men and women. Follow‐up analyses revealed that the association of promotion concerns with overly positive perceptions of own desirable traits was smaller for men than women and the association of prevention concerns with overly negative perceptions of own desirable traits was smaller for women than men; however, all of these associations were statistically significant in both groups, as were the overall indirect effects. For women, the positive direct effects of promotion concerns were significant for the consensus desirability of both chosen and non‐chosen partners and were larger than the effects for men. For men, the positive direct effects of promotion concerns were only significant for the consensus desirability of chosen partners and there were also significant negative direct effects of prevention concerns for chosen partners. Thus, there was clearer support that the Hui et al. (2020) findings extended to broader desirability among men than among women. Additional details of the gender effects can be found in the supplement (Figures S8 and S9).

### 3.3 Discussion

Study 2 replicated and extended Study 1 by showing further evidence that promotion concerns were associated with higher perception of one's own desirability whereas prevention concerns were associated with lower perception of one's own desirability (Scholer et al. 2014). These perceptions then again predicted how high people set their standards for the desirability of an ideal romantic partner which then, in this study, predicted the consensus desirability of the potential romantic partners participants chose to pursue for a further relationship (see also Shaw Taylor et al. 2011). Furthermore, these results held even when controlling for participants' self‐esteem and the consensus desirability of their own traits as rated by the potential partners with whom they interacted. This provided clearer evidence that the observed indirect effects of regulatory focus reflected overly positive or negative perceptions (see Hazlett et al. 2011) and interest in partners more or less desirable than one's own level of desirability rather than any associations of regulatory focus with actual desirability as a partner.

Beyond these indirect effects of regulatory focus through self‐perceptions, the results of Study 2 did not show as clear evidence for direct effects of promotion concerns on the consensus desirability of partners that were pursued (cf. Hui et al. 2020). Such direct effects did emerge among the men in the sample, but not among the women.

## 4 General Discussion

People differ in their willingness to prioritize pursuing highly desirable partners, whatever their own level of desirability. Going beyond previous research on the broad role of generally positive self‐concepts and histories of individual experience in seeking romantic partners in explaining these differences (e.g., Penke et al. 2007; Shaw Taylor et al. 2011), the present research explored how basic motivational concerns with growth (promotion) or security (prevention) might reflect additional self‐regulatory influences on the role of self‐evaluation in determining who prioritizes identifying and pursuing more normatively desirable partners.

Two studies supported the hypotheses that, consistent with more positive self‐perceptions associated with the eager mindsets adopted during promotion‐focused goal pursuit, promotion concerns predicted the identification and pursuit of more normatively desirable romantic partners, whereas, consistent with more negative self‐perceptions associated with the vigilant mindsets adopted during prevention‐focused goal pursuit, prevention concerns predicted the identification and pursuit of less normatively desirable romantic partners. Specifically, Study 1 illustrated that, independent of general levels of self‐esteem, stronger promotion concerns positively predicted and stronger prevention concerns negatively predicted overall evaluations of one's own desirability; these self‐evaluations then predicted greater or lesser importance placed on desirable traits for ideal partners, which then further predicted how much the specific individuals identified as current romantic interests over a seven‐month period were rated as possessing these traits. Study 2 replicated and extended Study 1 by directly showing that stronger promotion concerns predicted overly positive evaluations, and stronger prevention concerns predicted overly negative evaluations, of one's own desirability controlling for both self‐esteem and consensus ratings of such desirability by potential partners; these evaluations then predicted higher or lower prioritization of desirability in ideal partners, which, in turn, further predicted specific interest in further contact with potential partners from a speed‐dating event who were consensually rated higher in desirability.

Thus, in summary, promotion concerns were associated with more positive and prevention concerns were associated with more negative self‐evaluations, which then predicted preferentially seeking romantic partners with similarly higher or lower levels of desirability. These results extend the findings of Hui et al. (2020) by (a) demonstrating such influence is independent of the associations of regulatory focus with global self‐esteem, and (b) illustrating complementary mechanisms involving the indirect effects of promotion‐ or prevention‐focused self‐evaluations on the level of aspirations people choose for their ideal standards for romantic partners and their pursuit of such partners when attempting to initiate relationships. Furthermore, Study 1 also presented evidence for additional direct influences of promotion concerns on the reported traits of specific romantic interests, which suggests that general eager strategies of pursuing highly desirable partners associated with these concerns may also apply to more than a prioritization of physical attractiveness. Evidence for such direct effects on the consensually rated attractiveness of speed‐dating partners participants desired to see again was less clear in Study 2, although it did emerge within the sample of men in that study. Further research on the direct effects of promotion‐ or prevention‐focused goal‐pursuit strategies on evaluation of romantic partners beyond any influences on self‐evaluation is thus needed to further clarify the roles of these different types of mechanisms.

### 4.1 Limitations of the Present Findings

Before considering the broader contributions of the present research, we should note some important limitations. First, all the present evidence is correlational. Covariate analyses helped eliminate some prominent alternative explanations, and the longitudinal design of both studies helped eliminate concerns about reverse causality—that is, that attraction to and pursuit of more or less desirable partners influences perceptions of one's own desirability or leads to the formation of higher or lower romantic ideals—but stronger evidence would come from studies manipulating promotion or prevention concerns in the context of evaluating romantic partners (see e.g., Hui et al. 2020, 2013). Moreover, further experimental work is particularly necessary to confirm the robustness of the mediational pathways suggested by Figures 2 and 3 (see Bullock et al. 2010).

Also, like much research on romantic relationships (McGorray et al. 2023), the present sample of participants lacked diversity and exclusively included heterosexual college students. Although we have no strong theoretical reasons to expect different results among individuals of different ethnicities, in same‐sex relationships, or in older age groups, it is possible that college students are more likely overall to be interested in and eager to pursue romantic relationships, and further research is needed before confidently generalizing the current findings beyond such populations.

A more specific limitation of the studies reported here is that the evidence for overly positive or negative impressions of oneself presented in Study 2 was based on the consensus estimates of speed‐dating partners with whom participants had only brief interactions. Therefore, controlling for this consensus allowed us to eliminate the actual judged desirability of participants as a factor in the context of the specific interactions assessed, and to interpret participants' judgments of themselves as exaggerated in this context. However, it does not necessarily assess participants' “true” overall desirability as a romantic partner as it might be judged over the course of more prolonged interactions. Associations of regulatory focus with overall desirability within such interactions should thus be examined further in future research.

Finally, there was some indication of moderation of the reported results in both studies by gender. It is important to note that (a) the pattern of these results was not entirely consistent between Studies 1 and 2, and (b) the novel indirect effects tested here remained significant overall for both men and women in both studies. Therefore, no clear evidence of systematic gender differences was observed. Nevertheless, despite this lack of systematic moderation, the present studies were not designed to have high statistical power to detect such effects. Therefore, small influences of gender on the reported results could exist and should be further evaluated in future research.

### 4.2 Contributions to Research on Attraction and Partner Selection

Despite these limitations, the present research makes several notable contributions to the literature on preferences for highly desirable partners in romantic relationships. Motivational tensions exist between universal aspirations for more desirable partners and strategies of pursuing romantic partners with similar levels of desirability to maximize the likelihood of success. However, few studies have directly investigated specific mechanisms through which people might prioritize desirability versus similarity. The present research expands these mechanisms beyond simple variations in self‐perception and self‐worth (Beckage et al. 2009; Shaw Taylor et al. 2011) and introduces motivational perspectives on how such self‐perceptions might arise and be strategically employed.

In particular, Study 2 illustrated how overly positive or negative self‐perceptions associated with promotion or prevention concerns predicted the higher or lower standards people set for the romantic partners they envisioned and subsequently pursued. Regulatory focus theory describes how, when focused on promotion, people strategically adopt eager mindsets focused on positive expectations, whereas when focused on prevention, people strategically adopt more vigilant mindsets focused on negative expectations to increase their engagement in goal pursuit (Hazlett et al. 2011; Scholer et al. 2014). The current research examined such effects in terms of individual differences, but circumstances might arise where promotion or prevention concerns are generally higher overall and systematic increases in positive or negative expectations might influence the pursuit of relationship partners.

For example, when people are focusing more on the potential gains of forming new relationships, such as stages of life where they are especially interested in the excitement such relationships can provide, or when they move to a new environment where they have few existing connections, broad promotion concerns might be evoked and they may more generally adopt more positive self‐perceptions and relationship ideals, whatever their level of self‐esteem or how desirable they are typically seen by others. In contrast, when people are more strongly prioritizing the security a relationship might provide, such as stages of life where they feel more ready to “settle down” and build a stable partnership, they may more generally adopt less positive self‐perceptions and relationship ideals. Similarly, across cultures, relationship norms that emphasize the pursuit of romantic partners more in terms of excitement versus stability could also predict general preferences for higher or lower levels of aspiration and standards for normatively desirable traits in a partner. More systematic tests of such possibilities are important directions for future research.

### 4.3 Contributions to Research on Regulatory Focus and Close Relationships

Several other prior programs of research have examined the influence of promotion and prevention concerns on close relationship processes (for a review see Molden and Winterheld 2013). However, most of this research has focused on dynamics of trust, commitment, and support within established relationships (e.g., Bohns et al. 2013; Cortes et al. 2018; Finkel et al. 2009; Hui et al. 2013; Molden and Finkel 2010; Molden et al. 2009; Righetti et al. 2011; Winterheld and Simpson 2016). The present research extends previous findings by illustrating how regulatory focus may also influence initial attraction and early relationship development (see also Hui et al. 2020). The current findings provide preliminary evidence for the role of promotion and prevention concerns in the priority people place on the overall desirability of potential romantic partners based on the level of aspirations they develop from perceptions of their own desirability. Future research could continue to explore the implications of such concerns for how people select romantic partners and initiate relationships in several ways.

As alluded to earlier, one direction for additional study involves whether there are specific characteristics people with stronger promotion or prevention concerns value in a partner. Some research (Cortes et al. 2018; Hui et al. 2013) suggests that promotion concerns predict a prioritization of growth experiences within relationships (e.g., excitement and autonomy) whereas prevention concerns predict a prioritization of security experiences (e.g., stability and reliability). These priorities in the context of existing relationships could also inform the characteristics that people seek out in potential romantic partners. The supplement details exploratory analyses in subsets of the traits evaluated in the present studies that produced mixed results for such effects. Some evidence was found for differing associations of promotion or prevention concerns on self‐enhancement and ideal preferences on distinct traits in Study 1, but not in Study 2. However, these studies were not designed to evaluate a comprehensive set of traits that might uniquely appeal to individuals concerned with promotion or prevention, and further research along these lines may be warranted. Even if the broader literature provides mixed evidence that specific partner ideals directly lead to the pursuit of partners who possess related traits (da Silva Frost and Eastwick 2024; Sparks et al. 2020), such ideals can still have important effects on relationship initiation and partner satisfaction (da Silva Frost and Eastwick 2024).

Another direction for additional study involves the readiness and confidence with which people seek to initiate romantic relationships. Eager mindsets produced by promotion concerns may encourage proactive efforts to seek romantic partners and establish relationships. In contrast, vigilant mindsets produced by prevention concerns may encourage caution and extend how long it takes to initiate and establish relationships. Note, however, that the present results indicate that eager mindsets would not be expected to produce lower levels of aspiration or lesser standards in the desire to initiate a relationship, and these mindsets may instead sustain more proactive pursuit through higher ratings of one's own desirability and greater expectations of success. In contrast, vigilant mindsets may lead to more cautious pursuit through lower ratings of one's own desirability and reduced expectations of success. Thus, additional research could more directly examine how these types of influences play a role in successful relationship initiation.

Finally, the association of promotion concerns with identifying and pursuing more desirable partners may create both benefits and drawbacks. Although the eager engagement with and pursuit of more desirable partners by individuals with stronger promotion concerns could be more likely to ultimately produce relationships with such partners, their overly positive evaluations also open them up to greater and more frequent rejection by partners “out of their league”. Furthermore, even when individuals with stronger promotion concerns secure highly desirable partners, if their aspirations have exceeded their own level of desirability, it may be more difficult for them to maintain a stable relationship with this partner over time (Penke et al. 2007). These too are interesting directions for future research.

### 4.4 Conclusion

Although people universally want to be with highly desirable romantic partners, not everyone places the same priority on or is willing to risk rejection to pursue such partners. The research described here examined what motivates individuals who do accept this risk. Our findings illustrate how the eagerness and increasingly positive expectations about one's own value as a romantic partner arising from stronger self‐regulatory concerns with growth and advancement predict a greater desire for and pursuit of desirable partners and thus provide new insight into when and why people searching for a relationship might look past the reality of their own desirability.

## Author Contributions

**Eileen Z. Wu:** conceptualization, investigation, formal analysis, software, visualization, writing – original draft. **Daniel C. Molden:** supervision, formal analysis, writing – review and editing. **Paul W. Eastwick:** investigation, methodology, data collection, writing – review and editing.

## Funding

Preparation of this article was supported in part by the National Science Foundation (Grant BCS‐0951571). The opinions and conclusions expressed herein are those of the authors and do not necessarily reflect the opinions of the NSF.

## Ethics Statement

All studies received ethics approval from the Northwestern University Institutional Review Board.

## Conflicts of Interest

The authors declare no conflicts of interest.

## Supporting information


**Data S1:** jopy700043‐sup‐0001‐Supinfo.docx.

## Data Availability

All materials, original datasets, and analysis code are accessible at https://osf.io/6tfe7/?view_only=81e08b67a3f0412f80408e94b3eceeb4.

## Endnotes

^1^
There are special circumstances in which these eager or vigilant mindsets can lead to a different pattern of prioritization. When people have already experienced some loss and may restore themselves to their starting point, prevention concerns create a greater emphasis on eliminating the loss and more strongly motivate seizing any opportunities to do so, despite chances for further loss (Scholer et al. 2010). In contrast, when people have already experienced some gain and have further chances to increase it, promotion concerns create a greater emphasis on preserving the gain, and more strongly motivate passing up opportunities for further gains when it risks giving up the gains already made (Zou et al. 2014). However, because the present research is primarily focused on people's pursuit of new romantic relationships, these special cases are less applicable and do not feature in our theorizing.

^2^

*Sociosexuality—*the extent to which people prefer to pursue short‐term romantic relationships centered around casual sex versus more long‐term, committed relationships—is another variable that has shown some relationship to the overall desirability of partners people pursue, at least with respect to a subset of prominent traits people regard highly, such as physical attractiveness and faithfulness (see Simpson et al. 2004). To ensure this additional variable was not explaining any observed associations of regulatory focus with self‐evaluations or partner preferences, we report additional analyses in the supplement (Figures S4 and S10) that show including sociosexuality attitudes as a covariate does not change the statistical significance of the hypothesized direct associations of regulatory focus with self‐perceptions or indirect associations with perceptions of or consensus ratings for romantic interests.

^3^
Two traits that participants also rated, “dominant” and “passive”, were excluded from the analyses because self and ideal partner ratings of these traits would not necessarily be expected to have the same clear positive associations with romantic interest ratings across the full sample as the other unambiguously positive traits would. These exclusions were made a priori before any analyses had been conducted.

^4^
Alternative analyses involving multiple regressions to assess mediation produced consistent conclusions for promotion concerns, which showed a statistically significant indirect effect, but the indirect effects for prevention concerns were not significant. This latter result could reflect the reduced sensitivity of regression analyses that do not adjust for measurement error as compared to the present SEM approach (Wang and Eastwick 2020). Full details of the regression results are provided in the Table S3.

^5^
A meta‐analysis by Judge et al. (2002) suggested that self‐esteem is highly correlated with feelings of competence, achievement, and confidence in the form of self‐efficacy at *r* = 0.85, and we thus have treated these variables as conceptually equivalent in our analysis. However, for those who might question whether a measure of overall self‐worth adequately controls for broad feelings of competence and achievement that could also explain the observed self‐evaluations in the Figure S5, we present analyses of the effects of regulatory focus controlling for alternative measures of a personal sense of power (Anderson et al. 2012) and environmental mastery (Ryff and Keyes 1995). In these analyses, indirect effects of promotion and prevention concerns and direct effects of promotion concerns remain significant.

^6^
Note that the speed‐dating sample analyzed here is distinct from the speed‐dating sample featured in Finkel et al. (2009), which summarizes preliminary, unpublished analyses on the associations between regulatory focus, overall liking of and desire for speed‐dating targets, and the perceived availability of other desirable romantic alternatives. This other sample did not include the full regulatory focus questionnaire (Higgins et al. 2001), and further scrutiny of the measures of promotion and prevention concerns that were included showed that they did not form reliable, internally consistent indices. This ultimately made it difficult to clearly interpret any observed associations of regulatory focus in this other sample, which is why such analyses are not reported here.

^7^
Parallel multiple regression mediation analyses in Study 2 produced similar findings to Study 1: promotion concerns showed a significant indirect effect on pursued‐partner desirability through self and ideal traits, whereas prevention concerns did not show a significant indirect effect. Full results are provided in the Table S6.
