# Supplementary material for: Promotion‐ or Prevention‐Focused Self‐Evaluation and the Preferential Pursuit of More Desirable Romantic Partners
Source: J Pers. 2025 Dec 16;94(5):834–48. doi: 10.1111/jopy.70043 (PMC13540329; doi:10.1111/jopy.70043)
Supplement: Supplementary file 1 [file JOPY-94-834-s001.docx]

**Supplemental Materials**

**Table of Contents**

***Supplementary Analyses: Study 1***

Table S1. *Individual Associations between Self-perceived Traits and Desired Traits and between Desired Traits and Perceived Traits of Romantic Interests* ................................................. p. s4

Confirmatory Factor Analysis of Promotion, Prevention, and Self-Esteem Scales ............. p. s4

Post Hoc Adjustments to Improve Fit of Primary SEM Model .......................*.....*............... p. s5

Figure S1. *Perceptions of Desirable Traits of Romantic Interests as Predicted by Promotion and Prevention Concerns, Perceptions of One’s Own Desirable Traits, and Rated Importance of Desirable Traits when Seeking a Romantic Partner in An SEM Model with Post Hoc Adjustments (Study 1) ...............................................................................................................................* p. s6

Figure S2. *Men’s Perceptions of Desirable Traits of Romantic Interests as Predicted by Promotion and Prevention Concerns, Perceptions of One’s Own Desirable Traits, and Rated Importance of Desirable Traits when Seeking a Romantic Partner (Study 1) ....*......*...*........ p. s7

Figure S3. *Women’s Perceptions of Desirable Traits of Romantic Interests as Predicted by Promotion and Prevention Concerns, Perceptions of One’s Own Desirable Traits, and Rated Importance of Desirable Traits when Seeking a Romantic Partner (Study 1) ...............*...... p. s8

Figure S4. *Perceptions of Romantic Interests’ Desirable Traits as Predicted by Promotion and Prevention Concerns, Perceptions of One’s Own Desirable Traits, and Rated Importance of Desirable Traits when Seeking a Romantic Partner while Controlling for Sociosexuality*

*(Study 1) ........................*....................................................................................................... p. s9

Alternative Measures of General Perceptions of Competence and Success ........................ p. s9

Figure S5. *Perceptions of Romantic Interests’ Desirable Traits as Predicted by Promotion and Prevention Concerns, Perceptions of One’s Own Desirable Traits, and Rated Importance of Desirable Traits when Seeking a Romantic Partner while Controlling for Environmental Mastery and Dispositional Power (Study 1) ......................................................................................* p. s11

Analyses of Separate Trait Dimensions of Vitality-Attractiveness Versus Warmth-Trustworthiness..................................................................................................................... p. s11

Figure S6. *Perceptions of Desirable Vitality-Attractiveness (VA) Traits of Romantic Interests as Predicted by Promotion and Prevention Concerns, Perceptions of One’s Own VA Traits, and Rated Importance of VA Traits when Seeking a Romantic Partner*

*(Study 1) ..................................*............................................................................................. p. s14

Figure S7. *Perceptions of Desirable Warmth-Trustworthiness (WT) Traits of Romantic Interests as Predicted by Promotion and Prevention Concerns, Perceptions of One’s Own WT Traits, and Rated Importance of WT Traits when Seeking a Romantic Partner (Study 1) ......*.............. p. s15

Table S2. *Associations of Promotion and Prevention Concerns with Perceptions of One’s Own Desirable Traits, Importance of Desirable Traits in an Ideal Romantic Partner, and Perceived Desirable Traits of One’s Own Specific Romantic Interests in Multiple Regression Analyses (Study 1)* ............................................................................................................................... p. s16

Analyses of Indirect Effects Using Multiple Regression ..................................................... p. s16

Table S3. *Individual Multiple Regression Analyses Included in Tests of Indirect Effects of Promotion and Prevention Concerns (Study 1)* .................................................................... p. s18

***Supplementary Analyses: Study 2***

Table S4. *Individual Associations between Self-perceived Traits and Desired Traits, and between Desired Traits and Consensus Ratings of the Desirable Traits of Speed-Dating Partners Pursued for Future Contact ............................*.................................................................................... p. s18

Confirmatory Factor Analysis of Promotion, Prevention, and Self-Esteem Scale ............... p. s19

Figure S8. *Consensus Desirability Ratings of Partners Men Did or Did Not Pursue as Predicted by Promotion and Prevention Concerns, Perceptions of Their Own Desirable Traits and the Desirable Traits of an Ideal Romantic Partner* .................................................................... p. s20

Figure S9. *Consensus Desirability Ratings of Partners Women Did or Did Not Pursue as Predicted by Promotion and Prevention Concerns, Perceptions of Their Own Desirable Traits and the Desirable Traits of an Ideal Romantic Partner* ....................................................... p. s21

Figure S10. *Consensus Desirability Ratings of Partners Participants Did or Did Not Pursue as Predicted by Promotion and Prevention Concerns, Perceptions of One’s Own Desirable Traits and the Desirable Traits of an Ideal Romantic Partner Controlling for Sociosexuality*

*(Study 2)* ................................................................................................................................ p. s22

Analyses of Separate Trait Dimensions of Vitality-Attractiveness Versus Warmth-Trustworthiness..................................................................................................................... p. s23

Figure S11. *Consensus Vitality-Attractiveness (VA) Ratings of Partners Participants Did or Did Not Pursue as Predicted by Promotion and Prevention Concerns, Perceptions of One’s Own VA Traits and the VA Traits of an Ideal Romantic Partner (Study 2) .........*.............................. p. s25

Figure S12. *Consensus Warmth-Trustworthiness (WT) Ratings of Partners Participants Did or Did Not Pursue as Predicted by Promotion and Prevention Concerns, Perceptions of One’s Own WT Traits and the WT Traits of an Ideal Romantic Partner (Study 2) .........*....................... p. s26

Table S5. *Associations of Promotion and Prevention Concerns with Perceptions of One’s Own Desirable Traits, Desirable Traits of an Ideal Romantic Partner, and Consensus Ratings of Desirable Traits of Partners Participants Chose to Pursue in Multiple Regression Analyses ..................................................................................................*............................................. p. s28

Analyses of Indirect Effects Using Multiple Regression ..................................................... p. s28

Table S6. *Individual Multiple Regression Analyses Included in Tests of Indirect Effects of Promotion and Prevention Concerns (Study 2)* .................................................................... p. s30

**Supplementary Analyses: Study 1**

**Table S1**

*Individual Associations between Self-perceived Traits and Important Traits in a Partner, and between Important Traits and Perceived Traits of Romantic Interests*

| Trait | *r*  Self and Important | *r*  Important and Romantic Interest |
| --- | --- | --- |
| Physically attractive | .35*** | .38*** |
| Ambitious / Driven | .40*** | .34*** |
| Dependable | .47*** | .22*** |
| Confident | .26*** | .34*** |
| Exciting | .41*** | .31*** |
| Spontaneous | .46*** | .29*** |
| Supportive | .30*** | .26*** |
| Optimistic | .35*** | .33*** |
| Realistic | .39*** | .35*** |
| Creative | .47*** | .40*** |
| Patient | .13☨ | .30*** |
| Level-headed | .27*** | .38*** |

*Note*. The relatively equivalent associations observed for individual traits suggests that participants were not normatively weighing a specific or smaller collection of traits more heavily than others, which is further evidence for aggregating trait ratings into a single index of desirability. It is important to note that the raw correlations in the second column do not control for normative desirability biases and thus do not constitute evidence of matching effects between individuals’ preferred ideals and the perceived traits of desired partners (see Sparks et al., 2020).

☨*p* < .10, **p* < .05, ** *p* < .01, *** *p* < .001

**Confirmatory Factor Analysis of Promotion, Prevention, and Self-Esteem Scales**

To examine whether promotion and prevention concerns represented separate constructs distinct from self-esteem, three confirmatory factor models were created: the first model loaded all of the promotion, prevention, and self-esteem items onto one factor; the second model loaded promotion and self-esteem items on to one factor and loaded prevention items on to a second factor; the third model loaded promotion, prevention, and self-esteem items load on to three separate factors. The overall fit of each model was then compared through likelihood ratio χ^2^ tests. Results showed that the three-factor model had the best fit, χ^2^ = 3563, df = 3, *p* < .001 compared to the one-factor model; χ^2^ = 78.4, df = 2, *p* < .001 compared to the two-factor model, which provides evidence that promotion and prevention concerns did indeed represent separate constructs distinct from self-esteem.

**Post Hoc Adjusts to Improve Fit of Primary SEM Model**

To ensure the results of Study 1 were not biased by poor overall fit of the primary SEM model, we conducted a post hoc examination of sources of misfit using modification indices assessed with the lavaan R package. Several large sources of misfit reflected (a) content overlap within particular clusters of ratings for specific traits, (b) conceptual overlap between the same traits rated as a description of the self and an ideal romantic partner (e.g., rating how *warm* one sees themselves and how *warm* one sees an ideal partner), and (c) empirical overlap between specific ratings of one’s own traits and answers to specific items on the regulatory focus and self-esteem scales. Because these overlaps are theoretically reasonable and consistent with prior literature on self–ideal overlap (Klohnen & Mendelsohn, 1998) and correlated constructs, we conducted an additional analysis that added a set of single item-to-item residual covariances reflecting these relationships to the SEM model illustrated by Figures 1 and 2.

The results from this alternative model are presented in Figure S1. The model demonstrated acceptable fit (Hu & Bentler, 1999): CFI = .948, RMSEA = .080 (90% CI [.076, .083]). Importantly, the addition of the residual correlations did not alter the statistical significance of any of the results reported in the main body of the article. The specific code for adjusted model can be found in the full analysis code available at https://osf.io/6tfe7/overview?view_only=81e08b67a3f0412f80408e94b3eceeb4.

**Figure S1**

*Perceptions of Desirable Traits of Romantic Interests as Predicted by Promotion and Prevention Concerns, Perceptions of One’s Own Desirable Traits, and Rated Importance of Desirable Traits when Seeking a Romantic Partner in An SEM Model with Post Hoc Adjustments (Study 1)*

Note: Coefficients represent unstandardized estimates from latent variable regressions. Ratings of each trait of participants’ specific romantic interests were averaged across all of the individuals they nominated over the course of the study. Adjustments to the SEM model reported in Figure 2 were made from post hoc examination of the modification indices to guide the inclusion of additional item-item covariances.

☨*p < .10, *p < .05, **p < .01, ***p < .001*

**Figure S2**

*Men’s Perceptions of Desirable Traits of Romantic Interests as Predicted by Promotion and Prevention Concerns, Perceptions of One’s Own Desirable Traits, and Rated Importance of Desirable Traits when Seeking a Romantic Partner (Study 1)*

Note: Coefficients represent unstandardized estimates from latent variable regressions. Ratings of each trait of participants’ specific romantic interests were averaged across all of the individuals they nominated over the course of the study.

☨*p < .10, *p < .05, **p < .01, ***p < .001*

For men, promotion concerns had a significant positive indirect effect on the consensus desirability of the traits possessed specific romantic interests participants chose to pursue , z = 2.94, *p =* .003, *b*=.02, 95% CI [0.015, 0.074], whereas prevention concerns had a significant negative indirect effect on the desirability of these partners, z= -4.78, *p* < .001, *b* = -.006, 95% CI [-.010, -.004].

**Figure S3**

*Women's Perceptions of Desirable Traits of Romantic Interests as Predicted by Promotion and Prevention Concerns, Perceptions of One’s Own Desirable Traits, and Rated Importance of Desirable Traits when Seeking a Romantic Partner (Study 1)*

Note: Coefficients represent unstandardized estimates from latent variable regressions. Ratings of each trait of participants’ specific romantic interests were averaged across all of the individuals they nominated over the course of the study.

☨*p < .10, *p < .05, **p < .01, ***p < .001*

For women, promotion concerns had a significant positive indirect effect on perceptions of the desirable traits possessed by one’s specific romantic interests, *z* = 4.80, *p <* .001, *b=.*115, 95% CI [0.068, 0.162], whereas prevention concerns had a significant negative indirect effect on these perceptions, *z*= -6.67, *p* < .001, *b* = -.016, 95% CI [-.030, -.016].

**Figure S4**

*Perceptions of Romantic Interests’ Desirable Traits as Predicted by Promotion and Prevention Concerns, Perceptions of One’s Own Desirable Traits, and Rated Importance of Desirable Traits when Seeking a Romantic Partner while Controlling for Sociosexuality (Study 1)*

Note: Coefficients represent unstandardized estimates from latent variable regressions. Ratings of each trait of participants’ specific romantic interests were averaged across all of the individuals they nominated over the course of the study.

☨*p < .10, *p < .05, **p < .01, ***p < .001*

When controlling for sociosexuality attitude, promotion concerns had a significant positive indirect effect on perceptions of the desirable traits possessed by one’s specific romantic interests, *z* = 7.95, *p <* .001, *b=* 0.096, 95% CI [0.072, 0.120], whereas prevention concerns had a significant negative indirect effect on these perceptions, *z* = -6.94, *p* < .001, *b* = -.016, 95% CI [-.020, -.011].

**Alternative Measures of General Perceptions of Competence and Success**

To evaluate alternative strategies for controlling for any influence of general perceptions of competence and success beyond global self-esteem, we repeated the SEM analysis depicted in Figure 2 substituting measures of *environmental mastery* (Ryff & Keyes, 1995) and a *personal sense of power* (Andersen et al., 2012) for global self-esteem. Both measures had moderate zero-order correlations with promotion concerns, r = .57 and r = .58, respectively, *ps* <.001, and small zero-order correlations with prevention concerns, r = .15 and r = -.15, respectively, *ps* <.036. Environmental mastery is a three-item scale (𝛼=.62) that includes statements such as “in general, I feel I am in charge of the situation in which I live”. Personal sense of power is an eight-item scale (α = .88) that includes statements such as “I can get others to do what I want”.

As with the primary analyses reported in the manuscript, all analyses were performed using structural equation modelling (SEM) with latent variables, and all models were fitted using unweighted least squares (ULS). The results of these analyses displayed in Figure S5 indicate that promotion concerns had a significant positive indirect effect on reports of the desirable traits possessed by one’s specific romantic interests, *z* = 2.54, *p =.*026, *b*=.011, 95% CI [0.011, 0.089]. In contrast, prevention concerns had a significant negative indirect effect on these reports, *z* = -2.58, *p* = .01, *b* = -.005, 95% CI [-0.008, -0.001].

**Figure S5**

*Perceptions of Romantic Interests’ Desirable Traits as Predicted by Promotion and Prevention Concerns, Perceptions of One’s Own Desirable Traits, and Rated Importance of Desirable Traits when Seeking a Romantic Partner while Controlling for Environmental Mastery and Dispositional Power (Study 1)*

Note: Coefficients represent unstandardized estimates from latent variable regressions. Ratings of each trait of participants’ specific romantic interests were averaged across all of the individuals they nominated over the course of the study. ☨*p < .10, *p < .05, **p < .01, ***p < .001*

**Analyses of Separate Trait Dimensions of Vitality-Attractiveness Versus Warmth-Trustworthiness**

In analyzing the ideals people form for potential romantic partners, Fletcher et al. (1999) proposed three different types of characteristics: (a) Warmth-Trustworthiness/Intimacy-Loyalty attributes, (b) Vitality-Attractiveness/Passion attributes, and (c) Status-Resources attributes. According to [Fletcher et al. (1999)](https://www.sciencedirect.com/science/article/pii/S0022103118305961#bb0125), each of these categories “reflects a possible ‘solution’ to a specific barrier to successful reproduction” (p. 87) that existed in humans’ ancestral past and provides a systematic approach to evaluating romantic ideals. With this approach, even though attributes like *attractive* and *adventurous* are not colloquially synonymous, they both belong in the factor “Vitality-Attractiveness” because they can be used to identify a partner who is healthy and reproductively viable. To examine whether regulatory focus might differentially predict the types of traits that people perceive more highly in themselves or that they seek in a romatnic partner, we reanalyzed the data from Study 1 after creating separate indexes for these different factors. Because these studies were not designed specifically to assess the Fletcher et al. (1999) model, we did not have traits available to assess the Status-Resources dimension. For the other two dimensions, we categorized each trait we did measure using Tables 1 and 2 in Fletcher et al. (1999; see also Sparks et al., 2020).

**Measuring Self-Perceptions.** Participants’ ratings of their own traits on the vitality-attractiveness (VA) and the warmth (WT) dimensions were separately calculated. Based on Fletcher (1999), the index of VA traits for self-perceptions was the average of participants’ ratings of themselves as “physically attractive”, “ambitious / driven”, “confident”, “exciting”, and “spontaneous” (𝛼=.70). The index of WT traits for self-perceptions was the average of participants’ ratings of themselves as “dependable”, “supportive”, and “level-headed” (𝛼=.51). All these measures were answered on 1 (*Strongly disagree*) to 7 (*Strongly agree*) scales.

**Measuring Desired and Perceived Attractiveness in Romantic Interests.** At the in-person lab session, participants rated how much they agreed that the same sets of traits “matter[s] in your decision to start a romantic relationship with someone”. The desired VA traits (α = .64) and WT traits (α = .66) were averaged into separate indices. Whenever a new romantic interest was listed, whether at the first or any subsequent waves, participants rated their agreement that their romantic interests possessed these same traits, which were also averaged into separate VA (α = .69) and WT (α = .66) indices.

**Results and Discussion**

As with the primary analyses reported in the manuscript, all analyses were performed using structural equation modelling (SEM) with latent variables. All models were fitted using unweighted least squares (ULS). To examine the indirect associations of participants’ perceptions of their own VA or WT traits, these perceptions and importance ratings of either VA or WT traits in an ideal partner were treated as serial mediators predicting perceptions of desirable VA or WT traits in the specific romantic interests nominated over the seven months of the study, averaged across all of these individuals. The results of these analyses are displayed in Figures S6 and S7.

**Figure S6**

*Perceptions of Desirable Vitality-Attractiveness (VA) Traits of Romantic Interests as Predicted by Promotion and Prevention Concerns, Perceptions of One’s Own VA Traits, and Rated Importance of VA Traits when Seeking a Romantic Partner (Study 1)*

Note: Coefficients represent unstandardized estimates from latent variable regressions. Ratings of each trait of participants’ specific romantic interests were averaged across all of the individuals they nominated over the course of the study.

☨*p < .10, *p < .05, **p < .01, ***p < .001*

For VA traits, the overall fit of the model was comparable to the full model presented in the main body of the article, CFI = .94, RMSEA = .12. Promotion concerns had a significant positive indirect effect on perceptions of the desirable traits possessed by one’s specific romantic interests, *z* = 3.48, *p <* .001, *b*=.054, 95% CI [0.023, 0.084], whereas prevention concerns had a significant negative indirect effect on these perceptions, *z*= -4.71, *p* < .001, *b* = -.023, 95% CI [-.033, -.014]. Repeating these analyses including the post hoc adjustments described above does not change the significance of any direct or indirect effects of regulatory focus.

**Figure S7**

*Perceptions of Desirable Warmth-Trustworthiness (WT) Traits of Romantic Interests as Predicted by Promotion and Prevention Concerns, Perceptions of One’s Own WT Traits, and Rated Importance of WT Traits when Seeking a Romantic Partner (Study 1)*

Note: Coefficients represent unstandardized estimates from latent variable regressions. Ratings of each trait of participants’ specific romantic interests were averaged across all of the individuals they nominated over the course of the study.

☨*p < .10, *p < .05, **p < .01, ***p < .001*

For WT traits, the overall fit of the model was acceptable, CFI = .96, RMSEA = .10. For WT traits, promotion concerns had a non-significant indirect effect on perceptions of the desirable traits possessed by one’s specific romantic interests , *z* = 1.22, *p =* .22, *b* = .02, 95% CI [-0.01, 0.05], whereas prevention concerns had a significant positive indirect effect on these perceptions, *z* = 3.95, *p* < .001, *b* = .033, 95% CI [.016, .049]. Repeating these analyses including the post hoc adjustments described above does not change the significance of any direct or indirect effects of regulatory focus.

**Table S2**

*Associations of Promotion and Prevention Concerns with Perceptions of One’s Own Desirable Traits, Importance of Desirable Traits in an Ideal Romantic Partner, and Perceived Desirable Traits of One’s Own Specific Romantic Interests in Multiple Regression Analyses (Study 1)*

| Measure | Base Model | | | | Self-esteem and Gender Covariate Model | | |  |
| --- | --- | --- | --- | --- | --- | --- | --- | --- |
|  | Promotion | | Prevention | Promotion | | Prevention | Self-esteem |  |
|  | *b (SE), z* | | *b (SE), z* | *b (SE), z* | | *b (SE), z* | *b (SE), z* |  |
| Perceptions of own desirable traits | | .41 (.04), 9.78*** | -.01 (.03), -0.43 | .13 (.03), 4.18*** | | -.01 (.03), -0.54 | .17 (.03), 5.51*** | |
| Importance of desirable traits in an ideal partner | | .12 (.04), 2.73** | -.01 (.03), -0.36 | .12 (.06), 2.10* | | -.02 (.03), -0.58 | .00 (.04), 0.02 | |
| Perceived desirable traits of one’s specific romantic interests | | .11 (.04), 2.86** | .01 (.02), 0.32 | .09 (.05), 1.75☨ | | .01 (.03), 0.31 | .04 (.06), 0.58 | |

*Notes.* In the base model, promotion and prevention concerns were simultaneously included as predictors. The covariate model simultaneously added gender and self-esteem to this base model. Gender was coded as men = 0 and women = 1.

☨*p <* .10*, *p <* .05*, **p <* .01*, ***p <* .001

**Analyses of Indirect Effects Using Multiple Regression**

To replicate the analyses of the direct and indirect effects illustrated in the SEM model using traditional OLS multiple regression analyses, we first simultaneously predicted average ratings of one’s own desirable traits from promotion and prevention concerns, with self-esteem as a covariate, to estimate the coefficients from regulatory focus to the first mediator. We next predicted average ratings of the importance of desirable traits in a romantic partner from average ratings of one’s own desirable traits to estimate the coefficient from the first mediator to the second mediator. We finally predicted average ratings of perceptions of the desirable traits possessed by one’s specific romantic interests from promotion and prevention concerns and average ratings of the general importance of desirable traits in a romantic partner to estimate the coefficient of second mediator to the primary dependent variable and the remaining direct effects of regulatory focus. Indirect effects of regulatory focus on ratings of the traits possessed by romantic interests through self-perceptions and general importance ratings were estimated as the product of the (a) promotion or prevention coefficient in the first analysis, (b) self-rating coefficient in the second analysis, and (c) the general importance coefficient in the third analysis and tested using 5,000 bootstrap resamples to construct percentile 95% confidence intervals (Preacher & Hayes, 2008). Results of each individual regression analysis are presented in Table S3.

Results indicated that promotion concerns showed a significant indirect effect on pursued-partner traits through self and ideal traits, *b*= .051, 95% CI [.021, .095]. In contrast, the analogous indirect effect of prevention concerns was not significant, *b*= –.005, 95% CI [–.030, .017], These findings are consistent with the SEM results for the promotion indirect effect, but not the prevention indirect effect. This divergence likely reflects the fact that SEM adjusts for measurement error and intercorrelations among constructs, thereby allowing greater sensitively for detecting weaker associations. Similar to the SEM results, as shown in Table S3, promotion concerns showed a near significant direct effect and prevention concerns did not show a significant direct effect.

**Table S3**

*Multiple Regression Analyses Predicting Self Traits, Ideal Traits, and Partner Traits (Study 1)*

| **Path** | **Predictor** | **b (SE)** | **t** | **p** |
| --- | --- | --- | --- | --- |
| **DV = Own traits** | Promotion | 0.30 (0.072) | 4.19 | <.001 |
|  | Prevention | -0.03 (0.054) | -0.53 | 0.59 |
|  | Self-esteem | 0.40 (0.072) | 5.53 | <.001 |
| **DV = Ideal traits** | Own traits | 0.40 (0.064) | 6.25 | <.001 |
| **DV = Romantic interest traits (RI)** | Ideal traits | 0.44 (0.063) | 6.60 | <.001 |
|  | Promotion | 0.12 (0.064) | 1.86 | .06 |
|  | Prevention | 0.03 (0.063) | 0.52 | 0.61 |

**Supplementary Analyses: Study 2**

**Table S4**

*Individual Associations between Self-perceived Traits and Desired Traits in an Ideal Partner, and between Desired Traits in and Ideal Partner and Consensus Ratings of the Desirable Traits of Speed-Dating Partners Pursued for Future Contact*

| Trait | *r*  Self and Ideal | *r*  Ideal and Pursued Partner |
| --- | --- | --- |
| Physically attractive | .41*** | .12☨ |
| Sexy/Hot | .40*** | .12 |
| Good career prospect | .47*** | .27*** |
| Ambitious/ driven | .26*** | .29*** |
| Fun/ Exciting | .37*** | .13☨ |
| Funny | .21*** | .24*** |
| Responsive | .37*** | .15* |
| Dependable/trustworthy | .29*** | .10 |
| Friendly/nice | .32*** | .12☨ |
| Charismatic | .33*** | .26*** |
| Confident | .29*** | .26*** |
| Assertive | .39*** | .12 |
| Smart | .35*** | .09 |
| Intellectually sharp | .42*** | .01 |

*Note*. The relatively equivalent associations observed for individual traits suggests that participants were not normatively weighing a specific or smaller collection of traits more heavily than others, which is further evidence for aggregating trait ratings into a single index of desirability. It is important to note that the raw correlations in the second column do not control for normative desirability biases and thus do not constitute evidence of matching effects between individuals’ preferred ideals and the perceived traits of desired partners (see Sparks et al., 2020).

☨*p* < .10, **p* < .05, ** *p* < .01, *** *p* < .001

**Confirmatory Factor Analysis of Promotion, Prevention, and Self-Esteem Scales**

As in Study 1, to examine whether promotion and prevention concerns represented separate constructs distinct from self-esteem, three confirmatory factor models were created: the first model loaded all of the promotion, prevention, and self-esteem items onto one factor; the second model loaded promotion and self-esteem items on to one factor and loaded prevention items on to a second factor; the third model loaded promotion, prevention, and self-esteem items load on to three separate factors. The overall fit of each model was then compared through likelihood ratio χ^2^ tests. Results showed that the three-factor model had the best fit, χ^2^ = 2178, df = 3, *p* < .001, compared to the one-factor model; χ^2^ = 245.76, df = 2, *p* < .001, compared to the two-factor model, which provides evidence that promotion and prevention concerns represented separate constructs distinct from self-esteem.

**Figure S8**

*Consensus Desirability Ratings of Partners Men Did or Did Not Pursue as Predicted by Promotion and Prevention Concerns, Perceptions of Their Own Desirable Traits and the Desirable Traits of an Ideal Romantic Partner (Study 2)*

*Note*: Coefficients represent unstandardized estimates from latent variable regressions. A latent variable of the consensus ratings of participants’ own desirable traits by all speed-dating partners was also included as a covariate predicting participants’ perceptions of these traits (not depicted). Consensus ratings of speed-dating partners participants did or did not pursue romantic were averaged separately across all of the individuals in these categories.

☨*p* < .10, **p* < .05, ***p* < .01, ****p* < .001

For men, promotion concerns had a significant positive indirect effect on the consensus desirability of the traits possessed specific romantic interests participants chose to pursue , z = 2.23, *p =* .026, *b*=.026, 95% CI [0.003, 0.049], whereas prevention concerns had a significant negative indirect effect on the desirability of these partners, z= -2.17, *p* = .03, b = -.003, 95% CI [-.006, -.000].

**Figure S9**

*Consensus Desirability Ratings of Partners Women Did or Did Not Pursue as Predicted by Promotion and Prevention Concerns, Perceptions of Their Own Desirable Traits and the Desirable Traits of an Ideal Romantic Partner (Study 2)*

*Note*: Coefficients represent unstandardized estimates from latent variable regressions. A latent variable of the consensus ratings of participants’ own desirable traits by all speed-dating partners was also included as a covariate predicting participants’ perceptions of these traits (not depicted). Consensus ratings of speed-dating partners participants did or did not pursue romantic were averaged separately across all of the individuals in these categories.

☨*p* < .10, **p* < .05, ***p* < .01, ****p* < .001

For women, promotion concerns had a significant positive indirect effect on the consensus desirability of the traits possessed specific romantic interests participants chose to pursue , *z* = 2.23, *p <* .001, *b*=.010, 95% CI [0.001, 0.018], whereas prevention concerns had a significant negative indirect effect on the desirability of these partners, *z*= -2.28, *p* < .001, *b* = -.003, 95% CI [-.005, -.001].

**Figure S10**

*Consensus Desirability Ratings of Partners Participants Did or Did Not Pursue as Predicted by Promotion and Prevention Concerns, Perceptions of One’s Own Desirable Traits and the Desirable Traits of an Ideal Romantic Partner Controlling for Sociosexuality (Study 2)*

☨*p < .10, *p < .05, **p < .01, ***p < .001*

When controlling for sociosexuality attitude, promotion concerns had a significant positive indirect effect on perceptions of the desirable traits possessed by one’s specific romantic interests, *z* = 6.67, *p <* .001, *b=* 0.066, 95% CI [0.072, 0.120], whereas prevention concerns had a significant negative indirect effect on these perceptions, *z*= -6.03, *p* < .001, *b* = -.012, 95% CI [-.020, -.011].

**Analyses of Separate Trait Dimensions of Vitality-Attractiveness Versus Warmth-Trustworthiness**

As in Study S1, we reanalyzed the data from Study 2 after creating separate indexes for Vitality-Attractiveness or Warmth-Trustworthiness dimensions. Again, because these studies were not designed specifically to assess the Fletcher et al. (1999) model, we did not have traits available to assess the Status-Resources dimension. For the other two dimensions, we categorized each trait we did measure using Tables 1 and 2 in Fletcher et al. (1999; see also Sparks et al., 2020).

**Measuring Self-Perceptions.** Participants’ ratings of their own traits on the vitality-attractiveness (VA) and the warmth (WT) dimensions were separately calculated. Based on Fletcher (1999), the index of VA traits for self-perceptions was the average of participants’ ratings of themselves as “physically attractive”, “sexy / hot” ,“ambitious / driven”, “fun/ exciting”, “funny”, “confident” and “assertive (𝛼=.79). The index of WT traits for self-perceptions was the average of participants’ ratings of themselves as “responsive”, “dependable /trustworthy”, “friendly/ nice” (𝛼=.60). All these measures were answered on 1 (*Strongly disagree*) to 7 (*Strongly agree*) scales.

**Measuring Desired and Consensus Attractiveness in Speed Dates.** Participants rated how much the following traits described their ideal partner. The desired VA traits (α = .79) and WT traits (α = .63) were averaged into separate indices. The average consensus for the VA or WT traits of each participant was found to converge: VA (α = .92) and WT (α = .9).

**Results**

As with the primary analyses reported in the manuscript, all analyses were performed using structural equation modelling (SEM) with latent variables. All models were fitted using unweighted least squares (ULS). To examine the indirect associations of participants’ perceptions of their own VA or WT traits, these perceptions and ratings of desired VA or WT traits in ideal partner were treated as serial mediators predicting consensus ratings of traits calculated separately for the speed-dating partners that participants pursued (i.e., indicated they wanted to see again) and the partners they did not pursue (i.e. did not indicate they wanted to see again). As in Study 2, participants’ self-esteem was also included as a control variable predicting perceptions of one’s own VA/WT traits. In this study, consensus ratings of participants’ own VA/WT traits were also included as a control variable predicting perceptions of one’s own VA/WT traits (not pictured). Thus, the model coefficients represented the increased positivity or negativity of participants’ perceptions of their own traits beyond their general self-worth. The results of these analyses are displayed in Figures S11 and S12.

**Figure S11**

*Consensus Vitality-Attractiveness (VA) Ratings of Partners Participants Did or Did Not Pursue as Predicted by Promotion and Prevention Concerns, Perceptions of One’s Own VA Traits and the VA Traits of an Ideal Romantic Partner (Study 2)*

*Note*: Coefficients represent unstandardized estimates from latent variable regressions. A latent variable of the consensus ratings of participants’ own desirable traits by all speed-dating partners was also included as a covariate predicting participants’ perceptions of these traits (not depicted). Consensus ratings of speed-dating partners participants did or did not pursue romantic were averaged separately across all of the individuals in these categories. ☨*p* < .10, **p* < .05, ***p* < .01, ****p* < .001

For VA traits, the overall fit of the model was acceptable, CFI = .93, RMSEA = .09. Promotion concerns had a significant positive indirect effect on perceptions of the desirable traits possessed by one’s specific romantic interests, *z* = 3.26. *p <* .001, *b*=.019, 95% CI [0.007, 0.030], whereas prevention concerns had a significant negative indirect effect on these perceptions, *z*= -3.33, *p* < .001, *b* = -.005, 95% CI [-.008, -.002].

**Figure S12**

*Consensus Warmth-Trustworthiness (WT) Ratings of Partners Participants Did or Did Not Pursue as Predicted by Promotion and Prevention Concerns, Perceptions of One’s Own WT Traits and the WT Traits of an Ideal Romantic Partner (Study 2)*

*Note*: Coefficients represent unstandardized estimates from latent variable regressions. A latent variable of the consensus ratings of participants’ own desirable traits by all speed-dating partners was also included as a covariate predicting participants’ perceptions of these traits (not depicted). Consensus ratings of speed-dating partners participants did or did not pursue romantic were averaged separately across all of the individuals in these categories.

☨*p* < .10, **p* < .05, ***p* < .01, ****p* < .001

For WT traits, the overall fit of the model was acceptable, CFI = .96, RMSEA = .078. Promotion concerns had a non-significant positive indirect effect on perceptions of the desirable traits possessed by one’s specific romantic interests, *z* = 1.07, *p =* .28, *b*=.03, 95% CI [0.014, 0.115], whereas prevention concerns did not have a significant indirect effect on these perceptions, *z*= -.28, *p* = .78, *b* = -.00, 95% CI [-.002, .001].

Taken together, Studies S1 and S2 therefore provided consistent evidence that, as in the results aggregating across all the desirable traits measured, for VA traits, promotion concerns predicted optimistically higher estimates of one’s own attributes and prevention concerns predicted pessimistically lower estimates of one’s own attributes. These estimates then resulted in indirect effects on how one sets their ideals for potential partners regarding VA traits and the consensus ratings of the VA traits for the potential romantic partners identified and pursued. Although Study 1 provided some evidence that prevention concerns predicted more positive self-evaluations of WT traits, which then resulted in indirect effects on how one sets ideals for and who one identifies as a potential partner based on these traits, this did not replicate in Study 2. In contrast, more consistent with the aggregated results in both studies and the VA traits in Studies 1 and 2, promotion concerns again predicted more positive self-evaluations of WT traits, which then resulted in indirect effects on how one sets ideals for and who one identifies as a potential partner based on these traits. Thus, although there was some suggestion of the possibility of trait-specific indirect effects of regulatory focus, these results were not robust.

**Table S5**

*Associations of Promotion and Prevention Concerns with Perceptions of One’s Own Desirable Traits, Desirable Traits of an Ideal Romantic Partner, and Consensus Ratings of Desirable Traits of Partners Participants Chose to Pursue in Multiple Regression Analyses (Study 2)*

| Measure | Base Model | | Self-esteem and Gender Covariate Model | | | |  |  |
| --- | --- | --- | --- | --- | --- | --- | --- | --- |
|  | Promotion | Prevention | | Promotion | Prevention | Self-esteem | |  |
|  | *b (SE), t* | *b (SE), t* | | *b (SE), t* | *b (SE), t* | *b (SE), t* | |  |
| Perceptions of own desirable traits | .58 (.05), 10.93*** | -.07 (.03), -2.47* | | .41 (.06), 6.79*** | -.08 (.03), -2.23* | .17 (.03), 5.34*** | | |
| Desirability of ideal romantic partner traits | .25 (.06), 4.26*** | -.03 (.04), -0.70 | | .18 (.07), 2.53** | -.03 (.04), 0.76 | .07 (.05), 1.31 | | |
| Consensus trait desirability of pursued speed-dates | .09 (.03), 2.89** | -.02 (.02), -0.77 | | .03 (.03), 0.93 | -.03 (.02), -1.45 | .06 (.04), 1.37 | | |

*Notes.* In the base model, promotion and prevention concerns were simultaneously included as predictors. The covariate model simultaneously added gender and self-esteem to this base model. Gender was coded as men = 0 and women = 1. All analyses also controlled for the “objective” consensus ratings of one’s own traits by speed dates.

☨*p <* .10*, *p <* .05*, **p <* .01*, ***p <* .001

**Analyses of Indirect Effects Using Multiple Regression**

To replicate the analyses of the direct and indirect effects illustrated in the SEM model using traditional OLS multiple regression analyses, we first simultaneously predicted average ratings of one’s own desirable traits from promotion and prevention concerns, with self-esteem as a covariate, to estimate the coefficients from regulatory focus to the first mediator. We next predicted average desirability of traits in an ideal romantic partner from average ratings of one’s own desirable traits to estimate the coefficient from the first mediator to the second mediator. We finally predicted average consensus ratings of desirable traits of partners participants did pursue and partners participants did not pursue (in separate analyses) from promotion and prevention concerns and average desirability of traits in an ideal romantic partner to estimate the coefficient of the second mediator to the primary dependent variables and the remaining direct effects of regulatory focus. Indirect effects of regulatory focus on consensus desirability or partners participants did or did not pursue through self-perceptions and ideal partner ratings were estimated as the product of the (a) promotion or prevention coefficient in the first analysis, (b) self-rating coefficient in the second analysis, and (c) the ideal partner coefficient in the thrid analysis and tested using 5,000 bootstrap resamples to construct percentile 95% confidence intervals (Preacher & Hayes, 2008). Results of each individual regression analysis are presented in Table S6.

Results indicated that promotion concerns showed a significant indirect effect on consensus desirability of partners participants pursued through self and ideal traits, *b*= .046, 95% CI [.01, .098], but not on consensus desirability of partners participants did not pursue, *b*= -.012, 95% CI [-.056, .026], In contrast, the indirect effect of prevention concerns was not significant for partners participants either did pursue (*b*= -.009, 95% CI [-.025, .001]) or did not pursue (*b*= .002, 95% CI [-.006, .014]). As in Study 1, these findings are consistent with the SEM results for the promotion indirect effect, but not the prevention indirect effect, which again likely reflects the fact that SEM allows greater sensitivity for detecting weaker associations. As for direct effects, as shown in Table S6, similar to the SEM results, promotion concerns showed significant direct effects on both consensus desirability of partners participants did pursue and on partners they did not pursue. The direct effects of prevention concerns were not significant.

**Table S6**

*Individual Multiple Regression Analyses Included in Tests of Indirect Effects of Promotion and Prevention Concerns (Study 2)*

| **DV** | **Predictor** | ***b*** | **SE** | ***t*** | ***p*** |
| --- | --- | --- | --- | --- | --- |
| **Self traits** | Promotion | 0.515 | 0.062 | 8.27 | <.001 *** |
|  | Prevention | –0.103 | 0.051 | –2.01 | .046 * |
|  | Actual self (consensus) | 0.025 | 0.052 | 0.49 | .62 |
|  | Self-esteem | 0.341 | 0.059 | 5.81 | <.001 *** |
| **Ideal traits** | Self traits | 0.452 | 0.066 | 6.9 | <.001 *** |
| **Pursued partner traits** | Ideal traits | 0.198 | 0.076 | 2.61 | .01 * |
|  | Promotion | 0.165 | 0.078 | 2.13 | .035* |
|  | Prevention | -0.052 | 0.074 | -0.70 | .48 |
| **Not-pursued partner traits** | Ideal traits | -0.052 | 0.078 | -0.67 | .50 |
|  | Promotion | 0.162 | 0.081 | 2.00 | .048* |
|  | Prevention | -0.043 | 0.077 | -0.56 | .57 |
